# Revealing Hidden Biodiversity Footprints Embedded in Global Mining Supply Chains

**DOI:** 10.1021/acs.est.6c01834

**Published:** 2026-06-09

**Authors:** Yue Yu, Shuntian Wang, Livia Cabernard, Stephan Pfister

**Affiliations:** † Chair of Ecological Systems Design, Institute of Environmental Engineering, 27219 ETH Zurich, Zurich 8093, Switzerland; ‡ Sustainability Assessment of Food and Agricultural Systems, School of Management and School of Life Sciences, Technical University Munich, Freising 85354, Germany

**Keywords:** mining land use, biodiversity loss, spatially explicit assessment, supply chain impact mapping, multiregional input-output (MRIO) analysis

## Abstract

Driven by digitalization, infrastructure expansion, and the clean energy transition, global mining has grown rapidly over the past two decades, intensifying land-use pressure and sharpening trade-offs between resource extraction and biodiversity conservation. However, spatially explicit assessments of mining-related biodiversity impacts and the tracing of these impacts along mining supply chains remain limited. Here, we present a spatially explicit assessment framework that links local biodiversity intactness loss, expressed as mean species abundance, to global potential species loss, and we couple this framework with a multiregional input-output model to trace consumption-driven impacts along global mining supply chains. We find that biodiversity loss impacts associated with global mining land use are nearly twice as high as those of previous global estimates. Hotspots in Indonesia, New Caledonia, Australia, Brazil, and Peru account for 57% of the global mining-related biodiversity impacts. Coal, precious metals, nickel, iron, and copper extraction together contribute 82% of the total impact. Due to international trade, 77% of mining-related biodiversity footprints occur outside the countries of final consumption, with demand from China, Europe, Japan, and the United States accounting for 58% of the total footprints. Our results improve the transparency of biodiversity impacts embedded in global mining supply chains and support hotspot-oriented biodiversity conservation and supply chain governance.

## Graphical abstract

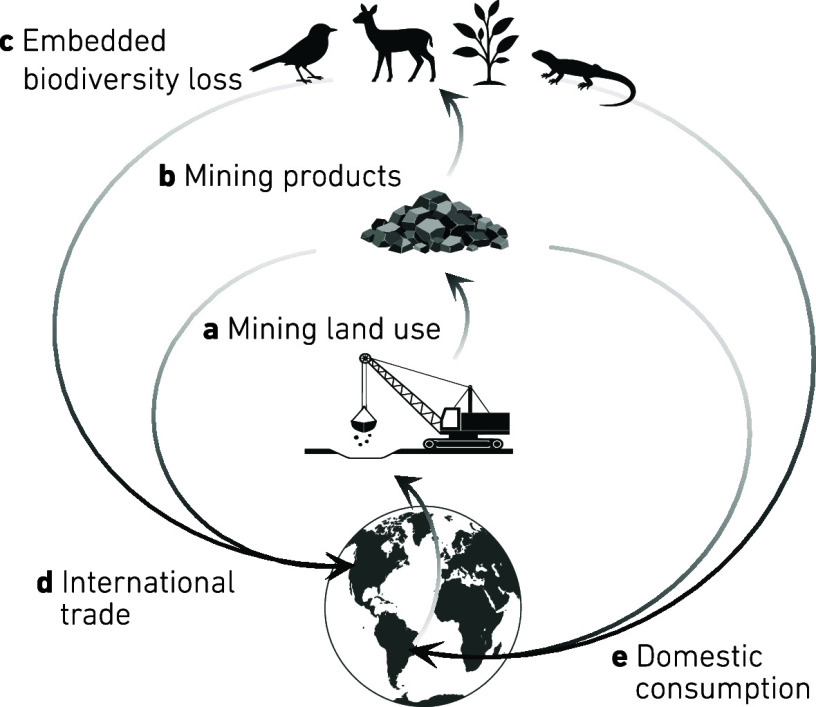

## Introduction

Driven by digitalization, infrastructure growth, and clean energy transitions,^1–4^ global mining activities have undergone rapid expansion over the past two decades. Global production volume surged from 11.3 billion tonnes in 2000 to 17.3 billion tonnes in 2020.^5^ This trend is projected to continue into the foreseeable future, particularly with the demand for critical minerals, which are necessary to achieve the net-zero emissions pathway.^6,7^ Rapid growth in demand for mining products has driven the expansion of mining land use, characterized by vegetation removal, topographic alteration, and habitat disruption.^8–12^ In recent years, delineating land used for mining has become feasible because of the enhanced availability of high-resolution satellite imagery and advancements in visual interpretation technologies. These developments have supported more effective monitoring of overlaps between mining operations and ecologically sensitive areas and have enabled the quantification of land-use-related impacts associated with these mining activities.^13–16^

Land use is regarded as the primary driver of terrestrial biodiversity loss.^17,18^ Mining areas frequently intersect with protected areas and key biodiversity areas, exacerbating tensions between resource extraction and conservation efforts.^1,10,11^ Mining land poses considerable threats to biodiversity due to habitat destruction and landscape fragmentation.^9,10,19^ For instance, it has been estimated that 3,264 km^2^ of forest was lost directly due to industrial mining between 2000 and 2019.^9^ Although mining has been a relatively minor driver of land-use-related biodiversity loss compared with other dominant sectors such as agriculture and forestry,^9,12,20,21^ its growing importance has been emphasized in biodiversity hotspots, including Indonesia,^9,22,23^ New Caledonia,^24–27^ Suriname,^9,28^ and Brazil.^8,9^

To improve the identification and traceability of land-use-related biodiversity loss from global mining activities, a spatially explicit assessment framework is essential. Such a framework can help identify hotspots and provide a more comprehensive understanding of mining-related biodiversity impacts. Previous studies have assessed deforestation caused by mining both within and beyond lease boundaries,^8,9,28^ whereas broader biodiversity impacts, particularly across multiple species groups, remain less well understood. Recent research has also applied characterization factors derived from species–area relationship models to quantify mining-related biodiversity loss.^12,20^ However, these assessments generally relied on country-average or ecoregion-specific characterization factors,^29^ thereby overlooking substantial intracountry and intraecoregion variation. Such limitations are particularly significant for mining because mining land use is typically more spatially fragmented and localized within countries and ecoregions than other land uses, such as agriculture. In addition, in previous mining-related biodiversity impact assessments, artisanal and small-scale mining activities have been poorly covered.^12,14,16^ Integrating updated mining land-use maps with high-resolution biodiversity data is therefore crucial for improving spatially explicit assessments of mining-related biodiversity impacts. Furthermore, globalization and international trade have increasingly enabled countries to externalize the environmental costs of resource extraction.^30–32^ Although environmentally extended multiregional input-output (MRIO) models have improved the tracing of consumption-driven land-use impacts across borders,^33–35^ their application remains constrained by coarse regional and sectoral resolution. Coupling spatially explicit biodiversity impact assessments with MRIO analysis that has been enhanced in both spatial and sectoral resolution is therefore necessary to better identify biodiversity footprints embedded in global mining supply chains.

In this study, we aim to address key research gaps by quantifying spatially explicit biodiversity loss embedded in global mining supply chains. The primary objectives of this study are to (1) develop a spatially explicit framework to assess biodiversity loss impacts due to global mining land use and (2) trace these biodiversity footprints within global mining supply chains. The updated global mining land-use map,^13^ which includes both large-scale mining and artisanal and small-scale mining, is used for the biodiversity impact assessment. We developed a spatially explicit framework that integrates local biodiversity intactness loss—accounting for land use, habitat fragmentation, and road disturbance—with global biodiversity importance to scale these local impacts to global potential species loss. The assessed mining-related biodiversity impacts are further integrated into a highly resolved MRIO database (i.e., Resolved EXIOBASE3 (REX3)^33^) to better understand biodiversity footprints and supply chain pathways linking producers to final consumers. The findings of this study are expected to promote sustainable practices in mining extraction by identifying hotspot regions and improving the transparency of biodiversity footprints along global mining supply chains.

## Materials and Methods

To assess biodiversity loss impacts embedded in global mining activities, we applied the methodological workflow, as shown in Figure 1. This spatially explicit assessment framework comprises two main components, namely land-use land-cover (LULC) allocation and assessment of biodiversity loss impacts embedded in global mining activities. An LULC map, incorporating mining land use as a separate category, was first derived. Subsequently, local biodiversity intactness and global biodiversity importance were assessed at 10-arc-second and 30-arc-second resolutions, respectively, to quantify spatially explicit biodiversity impacts associated with mining land use. Finally, impacts across all mine areas were aggregated to the country level and allocated to individual mining commodities based on the monetary values of the respective extracted quantities by country in 2019. Detailed information is provided in the following sections.

**Figure 1:**
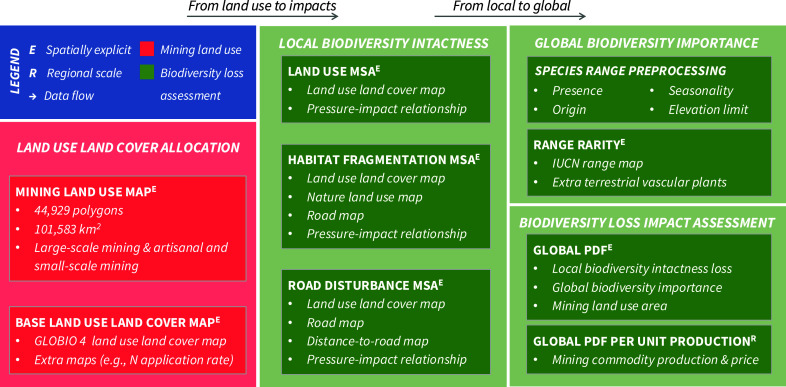
Spatially explicit framework to assess global mining-related biodiversity loss impacts. This framework comprises two main sections, namely land-use land-cover allocation (marked in red) and assessment of mining-related biodiversity loss impacts (marked in green). Superscripts E and R represent data resolution at spatially explicit and regional scales, respectively. The assessment workflow proceeds from mining-related land use to biodiversity impacts, and subsequently from local biodiversity intactness loss to the global potentially disappeared fraction of species. Abbreviations: N, nitrogen; MSA, mean species abundance; PDF, potentially disappeared fraction.

### Global Mining Land Use

In this study, the updated global mining land-use data set from Maus et al.,^13^ comprising 44,929 polygons and covering 101,583 km^2^ of both large-scale mining and artisanal and small-scale mining, was used to assess mining-related biodiversity loss. The data set was derived from visual interpretation of Sentinel-2 imagery for 2019. It includes common mining features such as open cuts, tailings dams, waste rock piles, water ponds, processing facilities, and other ground features related to mining activities, as well as isolated patches in between, which are generally considered to have reduced ecological function due to landscape fragmentation.^13,36,37^ Both above-ground and underground mining features visible in satellite images were included in the delineation. Different mining infrastructure types were not distinguished and were aggregated into a single mining land-use class. Active, inactive, and abandoned mines were likewise not differentiated as separate operational states in the mining land-use layer. Sites that still exhibited visible mining-related land-use features in the 2019 satellite imagery were included and treated as mining land use in the biodiversity assessment, whereas sites that had been revegetated or restored to the extent that mining features were no longer identifiable were not captured. Indirect mining-related land use, such as supply chain infrastructure and power plants supporting mining activities, or surrounding settlement growth induced by mining, was not included. To meet the input requirements of the biodiversity assessment model and align the spatial resolution with other data sets, the mining polygons were rasterized to a 10-arc-second resolution (∼300 m).

To characterize the spatial heterogeneity of land-use patterns and potential biodiversity impacts, it is necessary to move from coarse-grained maps or generic land-use models to spatially explicit LULC maps. As the fundamental input for the subsequent biodiversity assessment model, a LULC map at 10-arc-second resolution was derived based on the 2020 GLOBIO 4 LULC map,^38,39^ which is the closest available land-use year to the mining land-use map from Maus et al.^13^ In the predefined land-allocation order adopted for this study, the mining land-use data set from Maus et al.^13^ was given the highest priority, followed sequentially by urban areas, cropland, and finally forestry and pasture.^38,39^ We assigned the highest priority to mining because the data set from Maus et al.^13^ is a mining-specific land-use map derived from visual interpretation, whereas the background LULC map represents broader land-use classes generated through suitability allocation.^38,39^ Land-use intensity (i.e., intensive use and minimal use) was assigned to cropland areas based on a global nitrogen fertilization data set constructed by Adalibieke et al.^40^ A threshold of 100 kg/ha nitrogen application rate (eq 1) was applied to classify cropland intensity, considering the global average level in 2020:^38,40^

1 $$N_{\mathrm{all},j}=\frac{\overset{i=m}{\underset{i=1}{\sum }}\left(N_{i,j}\times {\mathrm{HA}}_{i,j}\right)}{\overset{i=m}{\underset{i=1}{\sum }}{\mathrm{HA}}_{i,j}}$$

where *N*_all,*j*_ (kg/ha) is the overall nitrogen fertilizer application rate for crops 1 to *m* within grid cell *j*, *N*_*i,j*_ represents the nitrogen fertilizer application rate of crop *i* within grid cell *j*, and HA_*i,j*_ represents the harvest area of crop *i* within grid cell *j*. The spatial distribution of nitrogen fertilizer application rates is depicted in Figure S1, which has been resampled using the nearest neighbor approach from its original resolution of 5-arc-minute to a finer 10-arc-second resolution to ensure consistency with the resolution of the LULC.

### Local Biodiversity Intactness Assessment

The GLOBIO 4 model was used to quantify local biodiversity intactness, expressed as mean species abundance (MSA).^39^ When assessing biodiversity intactness affected by global mining activities, land use, habitat fragmentation, and road disturbance were considered the main pressures, and plants and warm-blooded vertebrates (i.e., mammals and birds) were considered the affected taxa. For each species group, only the most relevant pressures were included—namely, land use for plants, and land use, habitat fragmentation, and road disturbance for warm-blooded vertebrates.^39^

For land use, pressure-impact relationships for both plants and warm-blooded vertebrates were represented by the remaining MSA values assigned to each LULC class (Tables S1 and S2).^38,39^ Habitat fragmentation impacts on warm-blooded vertebrates were quantified based on the size of natural patches, and the pressure-impact relationship was determined according to Schipper et al.^39^ Natural land cover and human land use were identified based on the GLOBIO 4 LULC classes, as shown in Table S3. Spatial data on road infrastructure were obtained from the Global Roads Inventory Project (GRIP) database,^41^ and main and secondary roads (i.e., road types 1, 2, and 3 in the GRIP database) were considered when calculating habitat fragmentation impacts. Additionally, noise, movement, and exhaust emissions, for example, can disturb habitats adjacent to roads and create road-effect zones that extend beyond the road itself.^38,39,41^ The pressure-impact relationship of road disturbance was determined based on the regression function between MSA and distance to the nearest road.^39^ The same GRIP database and road types were used to generate the distance-to-road map (Figure S2) and calculate road disturbance impacts.

The application of the GLOBIO 4 model in this study yielded four layers of residual MSA values at a 10-arc-second resolution: (1) plants and (2) warm-blooded vertebrates subject to land-use pressures, (3) warm-blooded vertebrates affected by habitat fragmentation, and (4) warm-blooded vertebrates subject to road-disturbance pressures. To aggregate MSA values across pressures, an overall MSA value was calculated by multiplying the pressure-specific MSA values,^39^

2 $${\mathrm{MSA}}_{g,j}=\overset{p=n}{\underset{p=1}{\prod }}{\mathrm{MSA}}_{p,g,j}$$

where MSA_*g,j*_ is the overall MSA for species group *g* (i.e., plants and animals) in grid cell *j* under pressures *p* from 1 to *n*, and MSA_*p,g,j*_ is the pressure-specific MSA. MSA values (eq 2) can be further aggregated to a larger scale of interest by calculating the mean values weighted by the area of the grid cells.

Local biodiversity intactness loss attributable to mining land use was quantified by comparing MSA values under the current mining scenario with those under a potential natural vegetation (PNV) baseline.^42^ The PNV map,^43^ which delineates the expected land-cover classes in the absence of human activities, was used as the natural reference (see PNV LULC description in Table S4). Mining sites were replaced with their corresponding PNV classes to estimate baseline biodiversity in the absence of mining activities. Table S5 provides the correspondence between the PNV LULC categories and those used in the GLOBIO 4 model.

### Global Biodiversity Importance Quantification

To ensure that environmental impacts across different geographical regions are comparable, it is crucial to quantify the scale of these impacts.^44,45^ For biodiversity loss, distinguishing between impacts that lead to local species loss and those that result in potential global species extinctions is essential.^44,46–50^ Range rarity, which combines species richness and endemism, was applied to identify the relative global biodiversity importance of the species assemblage present within a particular local area, thereby scaling the species loss from local to global scales. Regions with high range rarity are characterized by harboring a large number of species, species with limited global distributions, or both. For each species, the range rarity value for each grid cell is the proportion of the species’ range contained within that cell. These values can be summed across species to give the relative importance of each cell to the species occurring there.^51^

To calculate range rarity for animals, species ranges for four taxa (i.e., birds, terrestrial mammals, terrestrial reptiles, and amphibians) were obtained from the International Union for Conservation of Nature (IUCN) Red List.^52^ According to the Mapping Standards and Data Quality for the IUCN Red List Spatial Data, each species polygon is coded with presence (is/was the species in this area), origin (why/how the species is in this area), and seasonality (what is the seasonal presence of the species in the area) information.^53^ Range polygons were first filtered based on occurrence classes: “extant” presence; “native”, “reintroduced”, and “assisted colonization” origin; and “resident”, “breeding season”, “non-breeding season”, and “season occurrence uncertain” seasonality, following IUCN guidance.^51^ In addition, IUCN species elevation bounds^52^ were combined with the Global Multiresolution Terrain Elevation Data (GMTED)^54^ to further subtract the species range outside the elevation limits at the 30-arc-second resolution. Following the filtration process, the range maps for 10,997 bird species, 5,589 terrestrial mammal species, 7,651 amphibian species, and 9,345 terrestrial reptile species were utilized to compute the taxon-specific range rarity at 30-arc-second resolution, as outlined in eq 3.^51^ Aggregated animal range rarity in each grid cell was then calculated by summing the four taxon-specific range rarity layers, as outlined in eq 4.^51^

3 $${\mathrm{RR}}_{t,j}=\overset{s=k}{\underset{s=1}{\sum }}\frac{{\mathrm{RS}}_{t,s,j}}{\overset{j=l}{\underset{j=1}{\sum }}{\mathrm{RS}}_{t,s,j}}$$

4 $${\mathrm{RR}}_{\mathrm{animal},j}=\overset{t=4}{\underset{t=1}{\sum }}{\mathrm{RR}}_{t,j}$$

where RR_*t,j*_ is the range rarity of taxon *t* in grid cell *j*. For each taxon, species *s* from 1 to *k* were considered. RS_*t,s,j*_ is the range size of species *s*, taxon *t* in grid cell *j*. *l* is the total amount of grid cells at the global scale. RR_animal,*j*_ is the aggregated animal range rarity obtained by summing up the four taxon-specific range rarities, which is equivalent to summing the range rarity of all assessed species across the four taxa. It is worth noting that, as the recommended coordinate system for the IUCN Red List is the World Geodetic System 1984 (WGS84, EPSG: 4326), distortion of pixel sizes occurs from the equator to the polar regions. Instead of adjusting the distance that one degree of longitude covers, the range distribution maps were reprojected to an equal-area projection (EPSG: 6933) when calculating range size, ensuring pixel sizes were uniform across the range map, regardless of latitude.

To calculate range rarity for plants, we used a native range data set for red-listed vascular plants,^55^ including Maxent predictions for 27,208 species at 30-arc-minute resolution, to improve representativeness because the IUCN Red List provides limited spatial data for plants.^52^ For each plant species, the pixel-level probability^55^ was multiplied by the pixel area to estimate the range size that one cell encompasses. This value was then used to calculate the plant range rarity following eq 3. Plant range rarity was resampled to 30-arc-second resolution for spatial alignment with animal range rarity, although this resampling does not necessarily add fine-scale biological information beyond the native resolution of the underlying plant data set. Plant range rarity derived from IUCN data is shown in Figure S3 for comparison.

### Mining-Related Biodiversity Loss Assessment

Biodiversity loss due to land use can be assessed by multiplying the inventory (e.g., land occupation area) with corresponding characterization factors (e.g., species loss factors).^46,49^ Instead of utilizing ecoregion-specific or country-average global species loss factors from the UNEP-SETAC Life Cycle Initiative,^29^ spatially explicit characterization factors, derived by the multiplication of local biodiversity loss (i.e., ΔMSA) and global biodiversity importance (i.e., range rarity), were applied in this study:

5 $$CF_{g,j}=\Delta MSA_{g,j}\times RR_{g,j}\times N_{g}^{-1}$$

6 $$CF_{j}=0.5\times \left(CF_{\mathrm{plant},j}+CF_{\mathrm{animal},j}\right)$$

where, *g* refers to the animal or plant group, CF_*g,j*_ is the characterization factor for group *g* in grid cell *j*, indicating the global potentially disappeared fraction (PDF) of species per unit area of mining-related land use; ΔMSA_*g,j*_ is the change of local biodiversity intactness for group *g* in grid cell *j*; RR_*g,j*_ (ha^–1^) is the global biodiversity importance standardized by the corresponding area (ha) for group *g* in grid cell *j*; *N*_*g*_ is the number of assessed species for group *g*. When computing the characterization factor, RR_*g,j*_ was resampled to the 10-arc-second resolution to align with the resolution of ΔMSA_*g,j*_. Equal weight was given to animal and plant groups to derive the aggregated CF_*j*_.^47,56^ CF_*j*_ was applied to the mining area data obtained from Maus et al.^13^ to assess mining-related biodiversity loss impacts on a spatially explicit level, which could be further aggregated to a larger scale of interest (e.g., national level) by summing up the biodiversity loss impacts across grids. The global PDF values assessed in this study indicate potential global biodiversity loss impacts rather than the exact number of species extinctions that have already taken place or extinction probabilities, focusing on species evolution and genetic biodiversity.

To assess commodity-specific biodiversity loss, mining-related impacts were allocated to mined commodities at the country level by using 2019 production data. Several data sets provide global mining production data, including the World Mineral Statistics from the British Geological Survey (BGS) and the Metals and Mining Database from S&P Global Market Intelligence.^57,58^ For this study, mining quantities were directly adopted from the BGS^57^ because the SNL Metals and Mining Database covers a limited share of total production volumes relative to the BGS national accounts. Because globally consistent site-specific production and commodity information is unavailable for many mining polygons,^4^ impacts were allocated to extracted commodities using the top-down national allocation scheme proposed by Cabernard and Pfister.^12^ Country-level mining-related biodiversity loss impacts were allocated to different mining commodities based on the monetary values of the respective extracted quantities in 2019. Monetary values were calculated for each commodity and country by multiplying the production volumes from BGS^57^ with estimated commodity prices for 2019, as reported by the SNL Metals & Mining Database^58^ and the Mineral Commodity Summaries.^59^ The monetary allocation was applied to ensure consistency with the monetary transaction structure of the MRIO framework.^33^ Furthermore, given that polymetallic systems often produce coproducts with strongly differing physical quantities and economic values, monetary allocation can better reflect the revenue drivers of the extraction.^60,61^ Commodity-specific biodiversity loss, together with mining production data from BGS,^57^ was used to calculate the biodiversity impact associated with the production of each mining commodity at the country level.

### Supply Chain Impact Mapping

MRIO analysis is a top-down framework for tracing transactional flows (e.g., monetary values or physical units) and environmental accounts (e.g., greenhouse gas emissions) across global supply chains. MRIO analysis enables tracing flows of goods and services between regions and sectors, providing valuable insights into the links between production and consumption activities and their environmental impacts.^35,62,63^ Several MRIO data sets are currently available, including EXIOBASE3,^35^ Eora26,^62^ and GTAP,^63^ each with different regional and sectoral resolutions. The industry-by-industry version of EXIOBASE3 has high sectoral resolution (i.e., 163 sectors) but limited regional resolution, with only 44 countries and five aggregated “Rest of the World” regions (RoW regions). Because more than one-third of global land-use-related biodiversity impacts occur in these RoW regions,^20^ it is essential to couple EXIOBASE3 with other spatially resolved MRIO data sets to further identify biodiversity loss hotspots. In contrast, Eora26 is available at the national level but distinguishes limited sectors, with mining aggregated as a single sector. We used a highly resolved MRIO database (i.e., REX3),^33^ which was compiled following the approach of Cabernard and Pfister^34^ by merging the industry-by-industry version of EXIOBASE3 (v.3.8.2)^35^ and Eora26 (v.199.82).^62^ Data from BGS^57^ were integrated into REX3 to improve the data quality of the mining sectors. In the end, REX3 ultimately distinguishes 189 countries and 163 sectors, with time series from 1995 to 2022.

Supply chain impact mapping (SCIM) for global mining activities was conducted using the standard Leontief model.^64^ Mining-related biodiversity loss impacts (i.e., in global PDF) associated with one unit of production (i.e., in million euros per year) were derived by dividing the aggregated mining-related biodiversity loss impacts by the summed production values for each sector in each specific country, which were further incorporated into the REX3 database as the environmental extension. The year 2019 was selected for SCIM analysis because the assessment of mining-related biodiversity loss impacts was conducted based on the updated mining land-use data using 2019 satellite images.^13^ A 4D impact array was developed in this study with the dimensions of 163 × 189 × 189 × 163. The first and second dimensions represent production sectors and regions, respectively, while the third and fourth dimensions represent consumption regions and end-use sectors. For each dimension, the regions and sectors can be further aggregated for visualization. Detailed computational steps used to construct the 4D SCIM array are provided in Supporting Information Method S1.

## Results

### Local Biodiversity Intactness

Worldwide mining activities are currently responsible for an average global MSA decline of 0.047%, approximately equivalent to transforming 6 million ha of pristine habitat (i.e., MSA = 1) into degraded landscapes devoid of all original species (i.e., MSA = 0). This loss is equivalent to approximately 1.5 times the area of the country of Switzerland. Contributions to global MSA declines were analyzed at the country level for animals and plants, as shown in Figure 2a,b, respectively. Countries with extensive mining areas, notably Russia, China, Australia, the USA, and Indonesia (Figure S4), exhibit larger reductions in local biodiversity intactness for both species groups. Russia, which contains 12% of global mining areas, contributes the largest single share to global MSA reduction (14% for animals and 12% for plants). Collectively, the top 10 countries with the most extensive mining operations are responsible for 72% of animal-related MSA declines and 69% of plant-related MSA declines (see Supporting Information Result S1 for further results on MSA declines).

**Figure 2:**
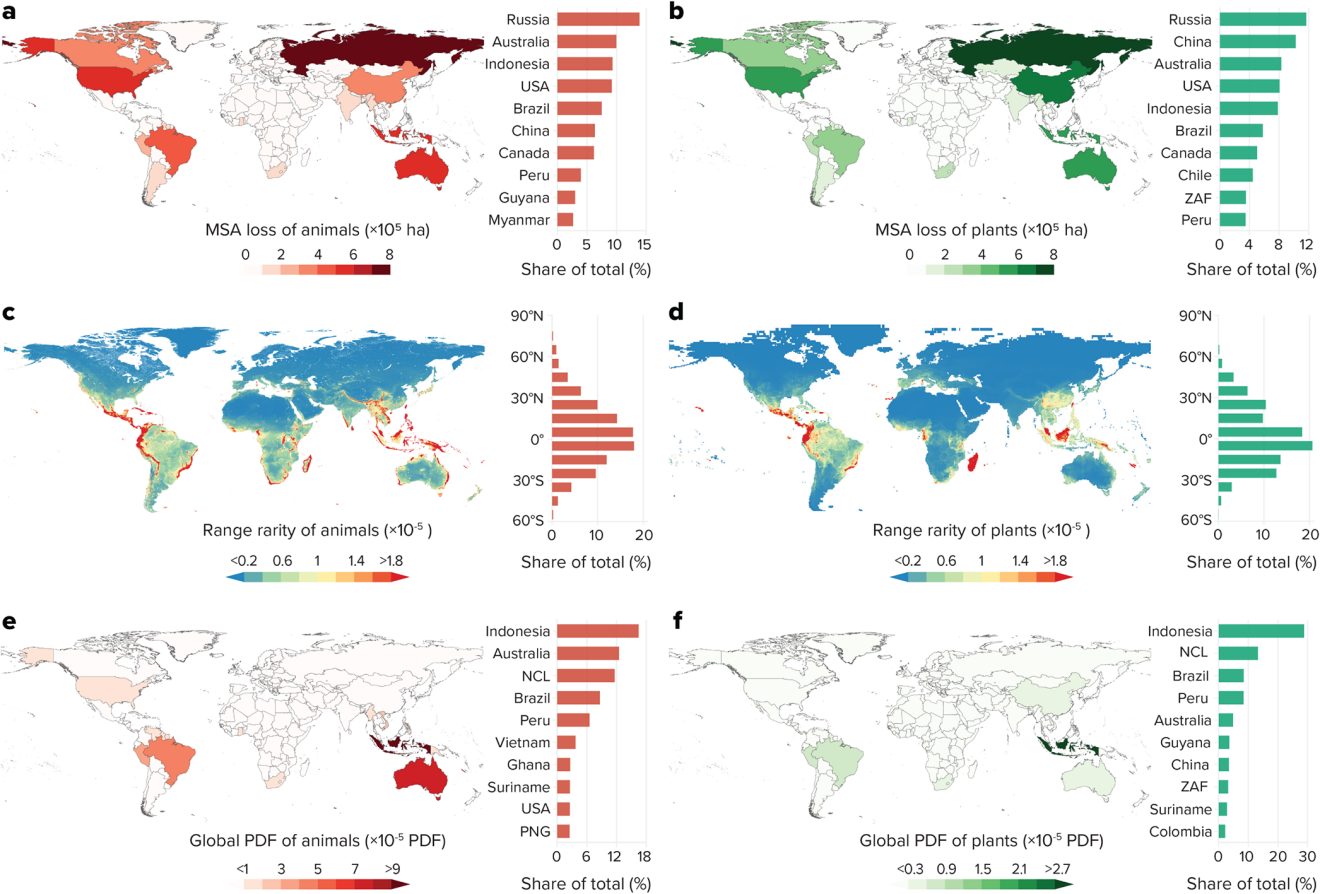
Land-use-related biodiversity loss impacts due to global mining activities. The resulting mining-related biodiversity loss represents (a, b) local biodiversity intactness loss, accounting for land use, habitat fragmentation, and road disturbance; (c, d) global biodiversity importance used to scale these local impacts; and (e, f) global potential species loss. Declines in local biodiversity intactness by country are expressed as mean species abundance loss for (a) animals and (b) plants, respectively. Contributions of the top 10 countries to global mean species abundance declines are presented in descending order. The spatial distribution of global biodiversity importance, expressed as range rarity, is shown at a 10-arc-second resolution for (c) animals and (d) plants. Adjacent bar charts display the latitudinal contributions to global range rarity. Detailed range rarity for four animal taxa (i.e., birds, terrestrial mammals, amphibians, and terrestrial reptiles) is shown in Figure S5. Global biodiversity loss impacts per country are expressed in the global potentially disappeared fraction for (e) animals and (f) plants. Contributions of the top 10 countries to global mining-related biodiversity loss are presented in descending order. Abbreviations: USA, United States of America; ZAF, South Africa; NCL, New Caledonia; PNG, Papua New Guinea; MSA, mean species abundance; PDF, potentially disappeared fraction.

### Global Biodiversity Importance

The importance of local intactness loss for global species loss, quantified by range rarity, was assessed for both animals and plants (Figure 2c,d, respectively). For animal species, range rarity was calculated for each taxon (Figure S5) and subsequently aggregated to an overall animal range rarity at a 10-arc-second resolution (Figure 2c). For plant species (Figure 2d), range rarity calculations were initially performed at a 30-arc-minute resolution and then resampled at the finer 10-arc-second resolution to align with the animal range rarity. Spatially explicit range rarity per grid cell spans several orders of magnitude and is influenced by differences in species richness and the range sizes of species present. Regions with high range rarity are characterized by hosting a considerable number of species, endemic species (i.e., species with restricted global distributions), or a combination of both. Common hotspots of high range rarity across all species groups are observed in South America, Southeast Asia, Madagascar, the Caribbean region, and the Himalayas. Conversely, regions with extreme temperatures, low precipitation, or limited habitat variation, such as the Sahara Desert, northern North America, and northern Eurasia, exhibit a relatively low range rarity. Mining activities conducted in regions with high range rarity may lead to irreversible species extinctions at the global scale, while in regions with lower range rarity, species loss may still occur locally, but there is a potential for restoration elsewhere.

### Hotspots of Global Mining-Related Biodiversity Loss

The spatial distributions of global mining land use and associated biodiversity loss impacts are shown in Figure 3. In total, global mining land use results in a potential biodiversity loss impact of 0.034% of global PDF. Hotspots, including Indonesia (18%), New Caledonia (12%), Australia (11%), Brazil (9%), and Peru (7%), account for 57% of the total impacts (Figure 4a), although these countries host only 26% of the global mining areas (Figure S4). It is worth noting that even small mining polygons in regions such as Indonesia (Figure 3b), Brazil (Figure 3e), and Peru (Figure 3f) can have high biodiversity loss impacts. Hotspot regions are located in areas with either severe local biodiversity intactness loss (Figure 2a,b), high biodiversity importance (Figure 2c,d), or both. Conversely, in regions such as Russia, China, and the USA, large mining areas exhibit comparatively low biodiversity loss impacts (see Supporting Information Result S2 for further examples). Additionally, spatially explicit biodiversity impacts were further aggregated to the country scale, with separate mining-related biodiversity loss estimates for animals and plants (Figure 2e,f). Indonesia, Australia, New Caledonia, Brazil, and Peru contribute to 56% of mining-related animal species loss and 64% of mining-related plant species loss, respectively (Figure 2e,f).

**Figure 3:**
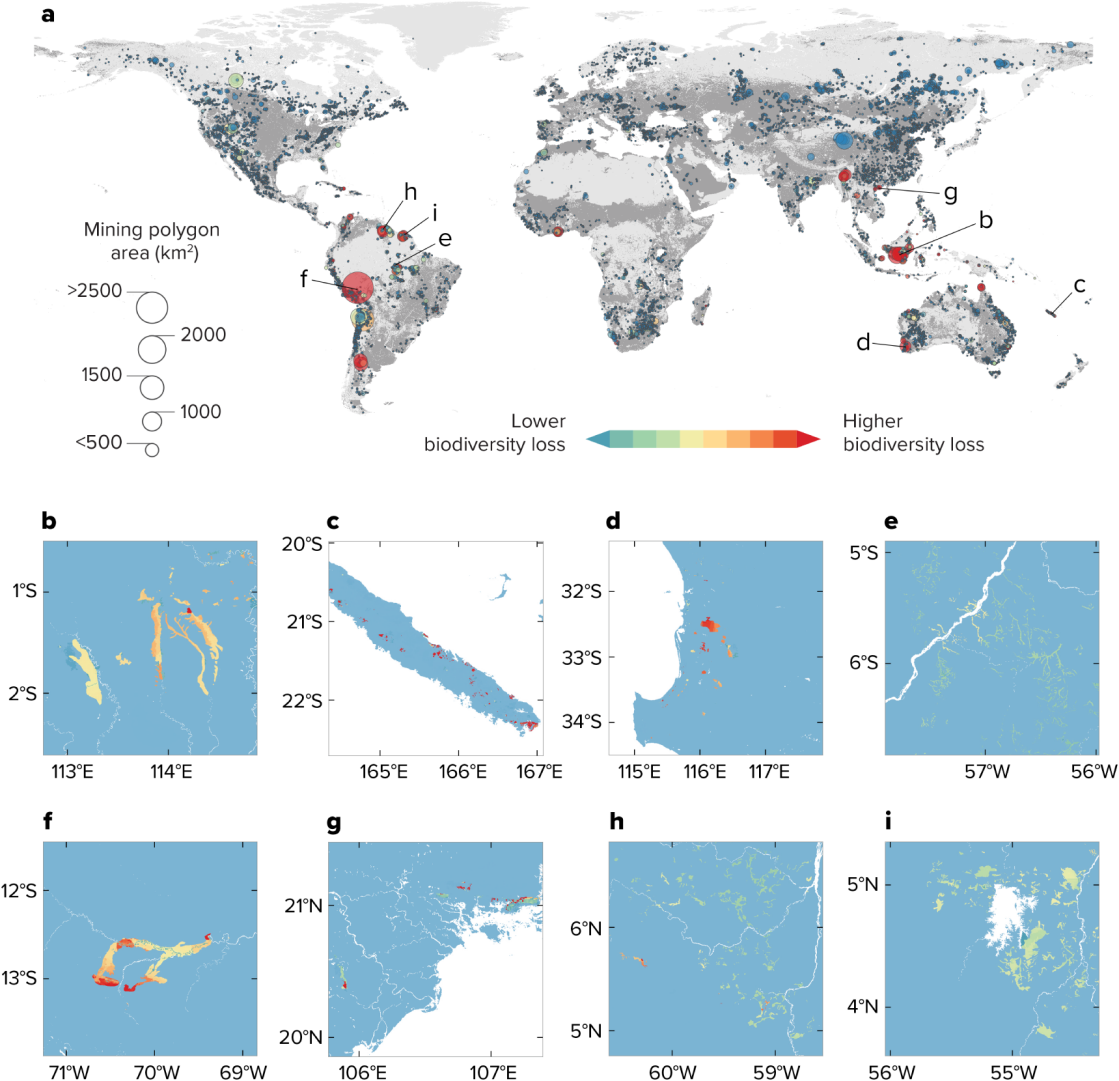
Hotspots of global mining-related biodiversity loss. (a) Global mining land-use area and mining-related biodiversity loss impacts are represented by the size and color of the circles, respectively. The center of each circle representing each mining site is located at the centroid of each mining polygon. The mining land-use distribution map is based on the data set by Maus et al.,^13^ comprising 44,929 polygons and 101,583 km^2^. See Supporting Information Result S4 and Figure S4 for detailed information on global mining land use. Natural land cover (marked in light gray) and human land use (marked in dark gray) are colored according to the LULC classification codes listed in Tables S1 and S2. (b–i) Hotspots of global mining land use and their spatially explicit biodiversity loss impacts in (b) Indonesia, (c) New Caledonia, (d) Australia, (e) Brazil, (f) Peru, (g) Vietnam, (h) Guyana, and (i) Suriname, which are the top eight countries contributing to the global mining-related biodiversity loss impacts. White areas in a–i represent water bodies.

**Figure 4:**
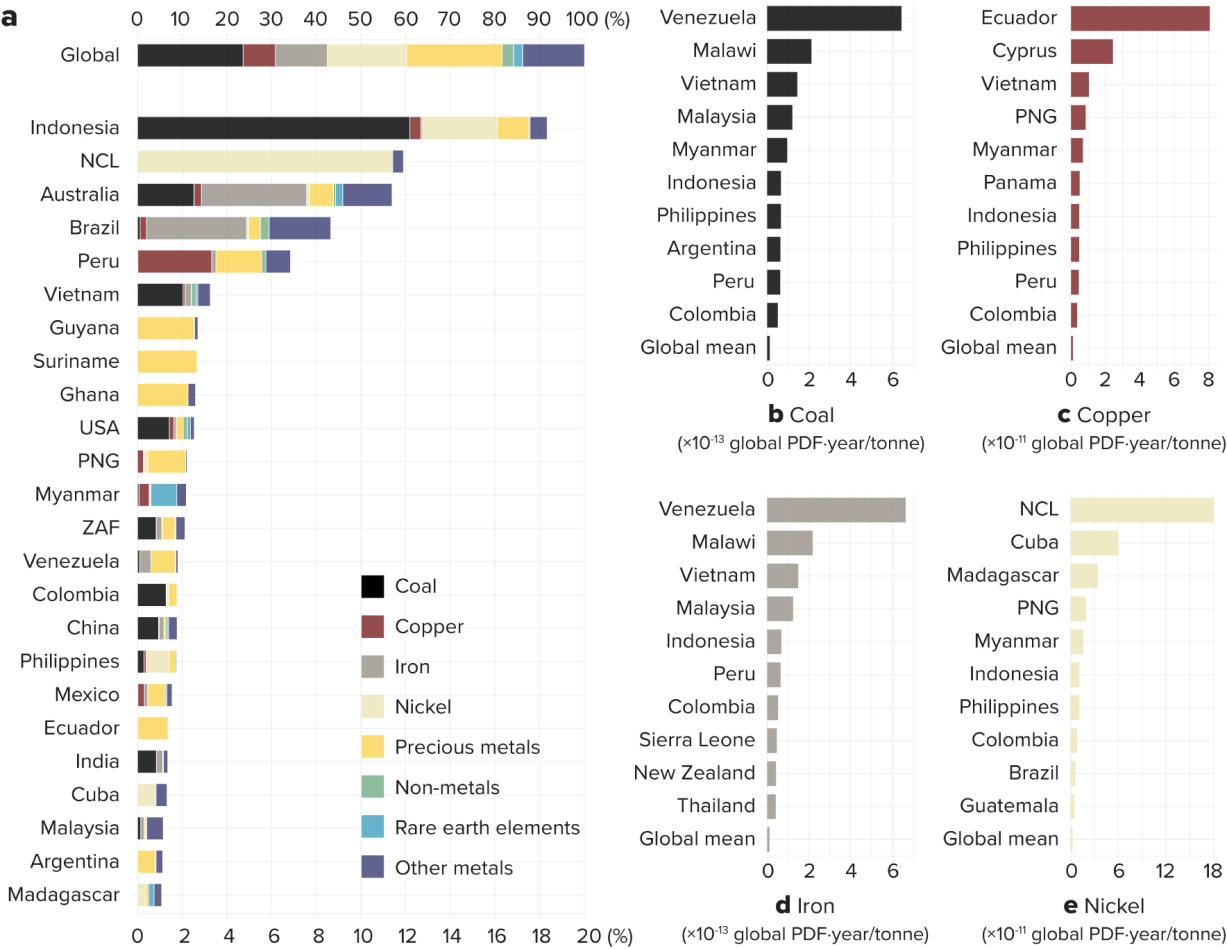
Global mining-related biodiversity loss impacts by country and commodity. (a) The sum of mining-related biodiversity loss impacts equals 0.034% of the global potentially disappeared fraction. All countries contributing more than 1% of the global mining-related biodiversity loss are listed in descending order. In total, these countries accounted for 93% of the global mining-related biodiversity loss. (b–e) Biodiversity loss impacts per unit production of (b) coal, (c) copper, (d) iron, and (e) nickel (expressed in global PDF · year/tonne) are listed for the top 10 countries, respectively. These four mining commodities together represented more than 60% of the global mining-related biodiversity loss. When calculating the global mean level of biodiversity loss impacts per unit production for each commodity, all countries producing that commodity were considered. Abbreviations: NCL, New Caledonia; USA, United States of America; PNG, Papua New Guinea; ZAF, South Africa; PDF, potentially disappeared fraction.

To further examine heterogeneity among mining footprints captured in the updated global mining land-use map,^13^ we grouped mining polygons into four footprint classes (i.e., < 0.1, 0.1–1, 1–10, and >10 km^2^) and analyzed their biodiversity impacts (Figure S6). These size classes should be interpreted as a spatial proxy for differences in mining footprint configuration rather than as an explicit classification of artisanal and small-scale mining versus large-scale mining. The analysis shows that polygons larger than 10 km^2^ account for only 3.9% of all polygons but contribute 64.7% of total mining area and 66.5% of total biodiversity impacts (Figure S6a). By contrast, polygons smaller than 1 km^2^ represent 75.4% of all polygons but contribute only 7.9% of total mining area and 5.2% of total impacts (see Supporting Information Result S3 for detailed analysis). These results indicate that both mining extent and regional ecological sensitivity shape the overall biodiversity burden.

Commodity-specific biodiversity loss impacts are shown for each country in Figure 4a. Overall, 82% of global mining-related biodiversity loss is attributed to the extraction of coal (24%), precious metals (21%), nickel (18%), iron (12%), and copper (7%). Coal mining in Indonesia and nickel mining in New Caledonia account for 12% and 11% of global mining-related biodiversity loss, respectively. Other biodiversity loss hotspots due to coal mining include Australia (2.5%), Vietnam (2%), the United States (1.4%), and Colombia (1.3%). Biodiversity loss impacts related to copper mining are largely located in Peru (3.3%), while most of the impacts associated with iron extraction occur in Australia (4.7%) and Brazil (4.5%). Indonesia is the second hotspot of nickel mining-related biodiversity loss, whereas Guyana, Suriname, Peru, and Ghana are hotspots for biodiversity loss impacts related to precious metal extraction.

Since the extraction of coal, nickel, iron, and copper contributes more than 60% of the total global impacts of mining, the biodiversity impacts per unit production of these mining commodities are further analyzed in Figure 4b–e. The top 10 countries shown in Figure 4b exhibit biodiversity loss impacts per unit of coal produced that are well above the global average, with Venezuela standing out with particularly high values. Although China, India, and the USA are the largest coal producers (together >60% of the global production), the biodiversity loss impacts per unit of production in these countries are below the global average. For copper (Figure 4c), impacts per unit produced are highest in Ecuador and Cyprus, while large biodiversity loss impacts are embedded in iron extraction in Venezuela, Malawi, Vietnam, and Malaysia (Figure 4d). New Caledonia and Cuba dominate the impacts per unit of nickel extracted (Figure 4e).

### Land-Use-Related Biodiversity Loss in Mining Supply Chains

Land-use-related biodiversity loss embedded in global mining supply chains is illustrated in a four-dimensional (4D) impact array, from (a) production sectors and (b) production regions to (c) consumption regions and (d) end-use sectors (Figure 5a–d). International trade drives 77% of the mining-related biodiversity footprint, which occurs in countries other than those of final consumption (Figure 5b,c). While China, Europe, Japan, and the USA account for only 6% of mining-related biodiversity loss from the production perspective, 58% of the global impacts were related to their consumption in 2019. This highlights that a vast majority of the mining-related biodiversity loss footprints of China (96%), Europe (91%), Japan (>99%), and the USA (81%) occur abroad (Figure 5f). In contrast, while 57% of global mining-related biodiversity loss impacts are induced in Indonesia, New Caledonia, Australia, Brazil, and Peru, only 10% of the global impacts are attributed to their territorial consumption. Exports, therefore, drive a significant share of mining-related biodiversity loss impacts in Indonesia (75%), New Caledonia (>99%), Australia (86%), Brazil (85%), and Peru (97%) (Figure 5e). Key trade flows contributing to mining-related biodiversity loss include exports from New Caledonia to Japan, as well as from Peru, Brazil, Indonesia, Australia, and other Asia–Oceania regions to China. China, the largest net importer of mining-related biodiversity impacts (Figure 5g), incurs 12% of its biodiversity loss footprint from coal mining in Indonesia, 9% from iron mining in Australia, and 8% each from copper mining in Peru and iron mining in Brazil. Meanwhile, Japan, as the second-largest net importer of mining-related biodiversity impacts (Figure 5g), has 65% of its mining-related biodiversity footprint attributed to nickel mining in New Caledonia.

**Figure 5:**
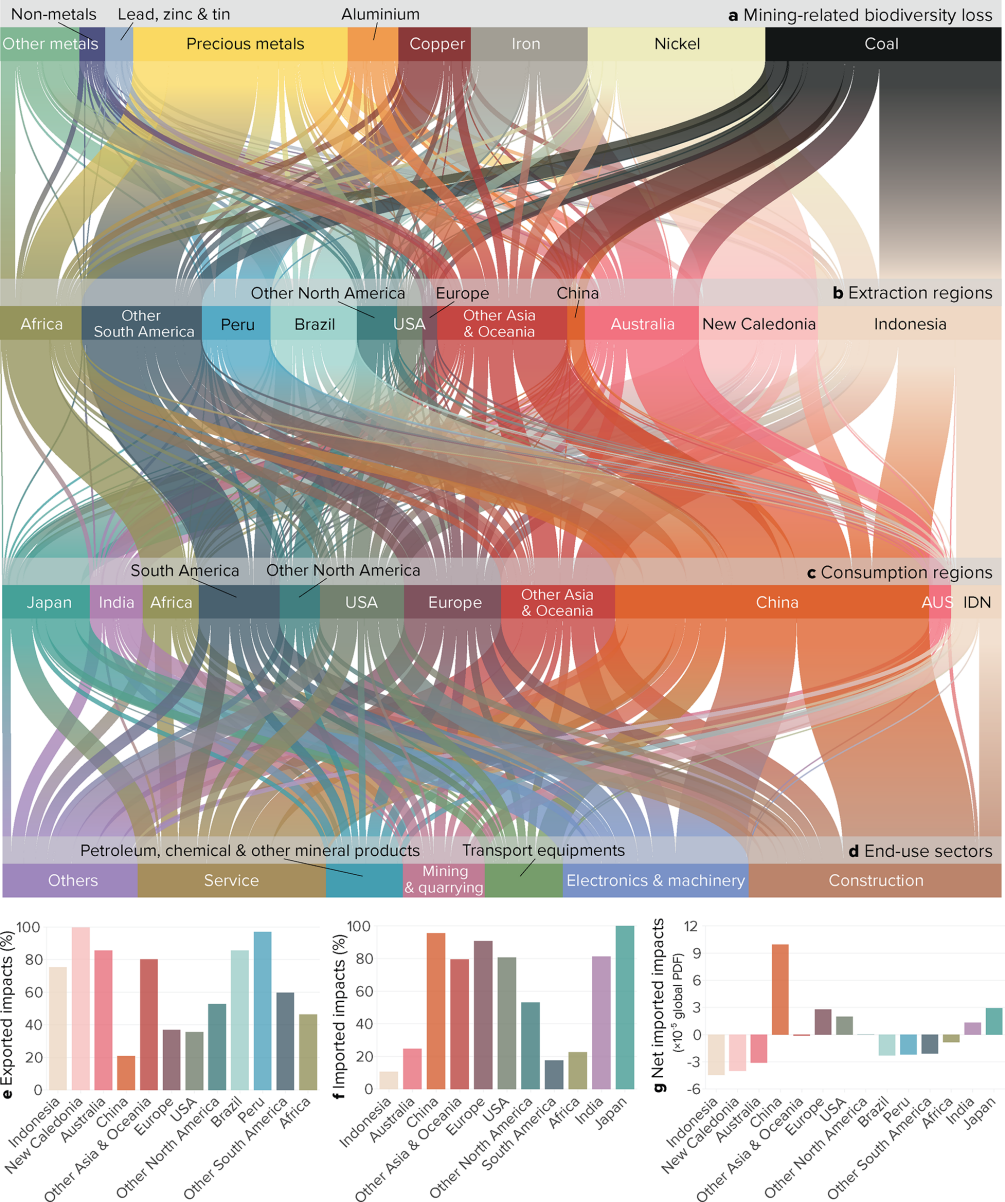
Supply chain analysis of mining-related biodiversity loss. (a–d) Land-use-related biodiversity loss embedded in global mining supply chains is illustrated in a 4D impact array, namely from (a) production sectors and (b) extraction regions to (c) consumption regions and (d) end-use sectors. (e–g) Shares of (e) exported and (f) imported mining-related biodiversity impacts are illustrated for the main extraction and consumption regions. Net imported mining-related biodiversity footprints (g) are shown for all the regions specified in the previous supply chain analysis (e**–**f). Countries that contribute to more than 5% of global mining-related biodiversity loss impacts, whether from production or consumption perspectives, are specified in the supply chain analysis, and the remaining countries are aggregated into world regions or subregions according to the Standard Country or Area Codes for Statistical Use (M49) from UNSD.^65^ Countries that are specified explicitly are not included in the aggregated world regions or subregions. Abbreviations: USA, United States of America; AUS, Australia; IDN, Indonesia; PDF, potentially disappeared fraction.

From the perspective of end-use sectors, 25% of the global mining-related biodiversity loss footprints are related to the global construction sector, with approximately half of these footprints resulting from consumption in China (Figure 5c,d). The construction sector is also the largest contributor to mining-related biodiversity loss in China, as well as in India and Indonesia, where it accounts for 42%, 29%, and 44% of their total footprints, respectively. Furthermore, services (e.g., trade, hospitality, public and social services), electronics, and machinery collectively contribute to 37% of the global mining-related biodiversity loss impacts, acting as the primary driver for the mining-related biodiversity footprints in Japan, the USA, and Australia, and the second-largest driver in China (Figure 5c,d). At the per capita level, consumption-based mining-related biodiversity loss footprints vary among regions (Table S6). Australia and Japan stand out with per-capita footprints approximately 6.7 and 5.3 times the global average, respectively. China and the USA, which are among the largest contributors to global mining-related biodiversity loss footprints, show per-capita footprints approximately 1.7 and 2.0 times the global average (Table S6). In contrast, regions including Africa, India, and other parts of Asia and Oceania show relatively low per-capita footprints. In Europe, all Eastern European countries fall below the global average on a per-capita basis, while most of the other European countries exceed the global average.

## Discussion

Our study provides a spatially explicit assessment of mining-related biodiversity loss impacts using updated mining land-use maps and identifies how consumption along global mining supply chains drives those impacts. We find that global mining land use results in 0.034% global PDF for the assessed species, which is almost twice as high as the estimate from the previous study.^12^ The top five mining countries—Indonesia, New Caledonia, Australia, Brazil, and Peru—together account for 57% of the total impact. Due to international trade, 77% of global mining-related biodiversity footprints occur in countries other than those of final consumption. More than 58% of those biodiversity impacts are attributable to just five countries, namely, China, Japan, the USA, India, and Indonesia.

### Spatial Hotspots of Mining-Related Biodiversity Loss

Mining-related biodiversity loss impacts have been assessed in previous studies using country-average or ecoregion-specific species loss factors from UNEP-SETAC.^12,20,29^ One major novelty of this study is the spatially explicit assessment framework that considers both local biodiversity intactness loss and global biodiversity importance. This multidimensionality of biodiversity is important because local ecological degradation does not necessarily translate proportionally into global biodiversity consequences, which also depend on the global biodiversity importance of the affected assemblages, particularly species richness and species range rarity.^50,66,67^ Previous research estimated global mining-related biodiversity loss at 0.02% global PDF,^12^ whereas the present study yields 0.034% global PDF. This primarily reflects methodological differences, including the use of the updated mining land-use data set,^13^ which substantially expands mining coverage and improves the representation of artisanal and small-scale mining, and the application of a more spatially explicit biodiversity assessment framework that enhances the spatial resolution and includes more anthropogenic pressures beyond direct land use. Although, at the global scale, mining represents a relatively small share of the global land-use-related biodiversity loss, which is dominated by agriculture (∼75%) and forestry (∼15%),^12,20^ its biodiversity burden remains highly relevant because impacts are spatially concentrated in ecologically sensitive regions and strongly embedded in international supply chains.^8,9,11,25,68^

Despite differences in spatial resolution and methodology, previous studies have consistently identified Indonesia, New Caledonia, Australia, and Brazil as biodiversity loss hotspots associated with global mining activities.^8,9,12^ Mineral and coal resource deposits in Indonesia substantially overlap with tropical rainforest, and Indonesia has experienced the largest deforested area due to industrial mining in tropical regions.^9,22,23^ New Caledonia and Australia have been widely recognized as biodiversity hotspots, where mining activities threaten numerous species.^24–26,69^ The global share of mining-related biodiversity impacts in Brazil increases from 4%^12^ to 8.6% in this study. One explanation is that artisanal and small-scale mining activities along rivers and water streams are more comprehensively covered in the updated mining land-use data set by Maus et al.^13^ than in the previous version by Maus et al.^14^ used by Cabernard and Pfister.^12^ Another explanation is that habitat fragmentation due to mining activities was not considered in the previous studies,^12,20^ while a large fraction of the mining sites in Brazil is located in natural areas, leading to higher risks of habitat fragmentation than in regions where most mining occurs in urban or secondary-vegetation land (Figure 3a).

In addition to the well-documented top contributors,^12^ new biodiversity loss hotspots such as Vietnam, Guyana, Papua New Guinea, Myanmar, and Ecuador emerged in this study (Figure 4a). This is due to both the expanded mining area data set^13,14^ and the high ecosystem values assigned to these areas compared with previous studies. For instance, the mapped extent of mining areas in Ecuador is more than ten times larger than in the previous version,^13^ which directly leads to higher biodiversity impacts. This expansion of mining land use was driven by the increased exploratory mining concessions issued by the Ecuadorian Ministry of Mining, most of which encroached on previously protected forests.^70^ In Guyana, the surge in gold extraction, boosted by artisanal and small-scale mining captured by the updated data set, has posed substantial biodiversity threats.^71^

### Commodity Hotspots of Mining-Related Biodiversity Loss

From the mining commodity perspective, coal, nickel, and precious metals account for more than half of global mining-related biodiversity loss impacts (Figure 4a), which is consistent with previous findings.^12^ It is worth noting that the allocation schemes in both studies are based on the monetary values of extracted metals in 2014 and 2019, respectively. Although this approach is consistent with the monetary MRIO framework and better reflects the revenue-driven nature of polymetallic mining than simple mass allocation, the relative contribution of each commodity to global mining-related biodiversity loss remains sensitive to price differences and temporal price fluctuations.

For individual commodities, an imbalance between the mining quantity and the biodiversity impact was observed. For instance, New Caledonia, which accounts for 8% of the global nickel production (Figure S7), contributes 64% of the nickel-related biodiversity impacts. The Koniambo nickel project, one of the largest nickel mining operations in the Northern Province of New Caledonia, is located in a region with high endemism (i.e., high range rarity), leading to overwhelming challenges in protecting fragile local biodiversity.^27^ In contrast, despite producing approximately the same amount of nickel as New Caledonia in 2019 (Figure S7f), Russia accounts for less than 1% of the nickel-related biodiversity impacts. A similar imbalance is observed in coal mining between Venezuela and China (Figure 4b). More broadly, the globally averaged biodiversity loss impacts per unit production of mining commodities may increase because of exploration activities driven by the energy transition, mining land expansion into protected areas, and declining ore grades.^1,11,72^

### Biodiversity Impacts Driven by International Trade

International trade is a major driver of mining extraction and the associated dislocation of environmental pressures.^12,20,68,73^ Overall, around 30% of global species threats have been linked to international trade in commodities, including minerals, fuels, and manufactured goods.^73^ In the mining sector, the share of global metal extraction connected to international supply chains increased from 63% in 1970 to 71% in 2020.^68^ This dislocation pattern is consistent with our finding that 77% of mining-related biodiversity footprints occur outside the countries of final consumption. Regions such as Indonesia, Australia, and New Caledonia bear a disproportionate share of total biodiversity impacts from the production perspective (Figure 5b), while major importing regions, including China, Europe, Japan, and the USA, account for a much larger share from the consumption perspective (Figure 5c). In practice, these outsourced mining-related biodiversity impacts tend to cluster where rich ore deposits, profitable extraction, and vulnerable ecosystems coincide, especially in tropical regions. International trade then shifts a substantial share of this damage from consuming to producing countries, often including biodiversity-rich producer regions in the tropics, while inconsistencies in environmental and supply chain governance limit effective biodiversity protection across global mining supply chains.

Given that current responsible or “green” mineral governance remains fragmented and still largely voluntary, more effective and better-coordinated governance across extraction, processing, trade, and final consumption is needed to reduce the displacement of biodiversity impacts through mineral trade.^74,75^ Potential measures include improving supply chain transparency to identify biodiversity hotspots, strengthening due diligence requirements, expanding recycling industries, and increasing the participation of local communities in mineral governance.^68,75^

### Limitations and Outlook

Our study assessed mining-related biodiversity impacts using the updated mining land-use map by Maus et al.^13^ Different land-use maps and delineation methods may yield different biodiversity loss hotspot patterns. Therefore, the assessment results should be interpreted together with a detailed description of the mining land-use map, and a standardized mapping methodology is needed in future research to improve study comparability (see Supporting Information Discussion S1 for further discussion of mining land-use mapping). In addition, the mining land-use map by Maus et al.^13^ was based on 2019 satellite data, and, for alignment, the retrieved mining production data were also from 2019.^57^ However, land-use distribution patterns and mining production volumes may have changed in recent years. For instance, the transition away from planet-warming energy sources toward clean-energy technologies has increased demand for energy transition minerals (ETMs),^1,2,4,7^ and the production of these ETMs has increased strongly in recent years (Figure S8),^59^ which may increase biodiversity loss risks at ETM mining hotspots, including nickel mining in Indonesia, the Philippines, and New Caledonia; lithium mining in Australia and Chile; cobalt mining in the Democratic Republic of the Congo; and rare earth element mining in China.

When deriving the spatially explicit biodiversity assessment framework, local biodiversity intactness loss due to global mining activities was first assessed and then upscaled to global potential species loss by weighting it by the global biodiversity importance. Spatial resolution differences between data sets may affect estimates of potential species loss in highly fragmented, high-biodiversity hotspots. Coarser biodiversity layers can smooth localized concentrations of range-restricted species, potentially underestimating impacts where small mining polygons overlap fine-scale biodiversity hotspots. Conversely, impacts may be overestimated where high biodiversity importance is assigned to a coarse grid cell, but the actual mining footprint occurs in a less suitable or less species-rich portion of that cell. Simply applying coarse extent-of-occurrence range maps at finer grid resolutions can create false spatial precision and misidentify biodiversity hotspots.^76^ Therefore, future improvements should rely on better biological information, such as locally validated occurrence records, atlas-based distributions, or habitat suitability models that better approximate fine-scale areas of occupancy, rather than on resampled coarse range maps alone.^76^ Furthermore, although the resulting MSA values are spatially explicit at 10-arc-second resolution, the pressure-impact relationships used are globally parameterized and do not necessarily capture biome- or region-specific differences in ecosystem sensitivity.^39^ This may lead to over- or underestimation of local biodiversity intactness loss in ecosystems whose responses differ from the global average. In addition, the current framework does not fully represent the breadth of biodiversity potentially affected by mining because several other species groups, such as freshwater species, fungi, and other less well-mapped taxa, are not explicitly included. Increasing taxonomic diversity and geographic representativeness would allow more species affected by global mining activities to be identified.^47,77,78^

It is worth noting that indirect biodiversity loss impacts from mining-supporting activities outside the mapped mining polygons of Maus et al.^13^ were not quantified. Previous studies have shown that mining can drive substantial off-site deforestation and biodiversity loss through transport and processing infrastructure, road expansion, settlement growth, and associated demand for land, timber, and fuelwood.^8,9^ Higher total biodiversity impacts associated with mining land use would be expected if indirect impacts were considered. Developing globally consistent methods to attribute such indirect impacts to mining remains an important priority for future research. In addition, the environmental impacts of mining extraction can occur over long distances beyond on-site land use.^10,70,79^ Recent global evidence suggests that mining may affect extensive freshwater conservation priority areas, and mining-derived dissolved and suspended contaminants can be transported and accumulated downstream, with detrimental impacts on stream biodiversity across multiple taxa.^79–81^ Furthermore, postclosure ecological recovery was not modeled explicitly. The present framework is based on the visible mining land-use footprint in 2019,^13^ and therefore captures the residual biodiversity impacts associated with the observed land-cover state rather than a dynamic trajectory of degradation and recovery. Incorporating recovery functions in future work would require temporally explicit information on mine closure and rehabilitation status, together with empirically grounded biodiversity recovery trajectories across ecosystems and taxa.

Because more than half of the global mining areas (56%) have no production information,^4^ commodity-specific impacts were allocated top-down based on monetary value in this study. Even for those mining areas with available production data, comining of multiple commodities (e.g., copper and gold) complicates the attribution of land-use impacts to specific minerals. A more refined bottom-up allocation based on mine-level disturbed area and actual extraction volumes would be highly valuable in future research if globally consistent data become available to link individual mining polygons to mine-specific production volumes, mining methods, and comining information. Such improvements would help better attribute the mining land-use footprint and associated biodiversity impacts to individual commodities.

## Supplementary Material



## Notes

Further information related to this study is presented in the Supporting Information. Supplementary methods on the construction of the 4D SCIM array (Method S1); additional results on local biodiversity intactness loss (Result S1); further examples on hotspots of global mining-related biodiversity loss (Result S2); polygon-level biodiversity impacts across mining footprint size classes and region groups (Result S3); additional information on global mining activities (Result S4); further discussion of global mining land-use mapping (Discussion S1); additional information provided as supporting figures (Figures S1–S8); and additional information provided as supporting tables (Tables S1–S7) (PDF)
