# Supplementary material for: Revealing Hidden Biodiversity Footprints Embedded in Global Mining Supply Chains
Source: Environ Sci Technol. 2026 Jun 9;60(24):17234–46. doi: 10.1021/acs.est.6c01834 (PMC13296505; doi:10.1021/acs.est.6c01834)
Supplement: Supplementary file 1 [file es6c01834_si_001.pdf]

Revealing hidden biodiversity footprints embedded in global mining supply chains

Yue Yu[1,*], Shuntian Wang[1], Livia Cabernard[2] and Stephan Pfister[1]

[1]Chair of Ecological Systems Design, Institute of Environmental Engineering, ETH Zurich, 8093, Zurich, Switzerland

[2]Sustainability Assessment of Food and Agricultural Systems, School of Management and School of Life Sciences, Technical University Munich, 85354, Freising, Germany

[*]Email: yueyuy@ethz.ch

Supplementary Information

Number of pages: 26

Number of text sections: 6

Number of figures: 8

Number of tables: 7

SI Methods

Method S1. Construction of the 4D SCIM array

The supply-chain analysis was conducted using Resolved EXIOBASE Version 3 (i.e., REX3[1]), which distinguishes 163 sectors and 189 countries or regions. Following the Supply Chain Impact Mapping (SCIM) approach[2-5], mining-related biodiversity impacts were traced from extraction to final consumption through a 4D impact array. In this study, the four dimensions represent: (1) extracted commodity, (2) extraction region, (3) consumption region, and (4) end-use sector.

First, country-level mining-related biodiversity loss impacts were allocated to extracted commodities based on the monetary value of production. The monetary allocation was applied to be consistent with the monetary transaction structure of the multiregional input-output (MRIO) framework[1]. Also, given that polymetallic systems often produce co-products with strongly differing physical quantities and economic values, monetary allocation can better reflect the revenue driver of the extraction[6,7]. This yielded a biodiversity impact extension $B_{m,r}$ for each extracted commodity m in extraction region r, expressed in global potentially disappeared fraction (PDF). The corresponding direct impact intensity was calculated as outlined in Eq. S1.

$$f_{m,r} = \frac{B_{m,r}}{q_{m,r}}, \qquad \text{(S1)}$$

where $q_{m,r}$ is the monetary output of commodity m in extraction region r in REX3[1]. The resulting coefficient $f_{m,r}$ represents the mining-related biodiversity impact per unit of monetary output for each extracted commodity-region pair.

The REX3 transaction matrix was then used to calculate the technical coefficient matrix A and the Leontief inverse in Eq. S2.

$$L = (I - A)^{-1}, \qquad \text{(S2)}$$

For each consumption region c and end-use sector e, we isolated the demand associated with that specific region-sector combination and used the Leontief inverse to calculate the total output required to satisfy it.

$$Z = L \times \hat{y}, \qquad \text{(S3)}$$

The resulting vector Z gives the total direct and indirect output across the global economy required by that selected region-sector combination.

The element $z_{m,r}^{c,e}$ denotes the corresponding element of Z, representing the monetary output of extracted commodity m in extraction region r required, directly and indirectly, by end-use sector e in consumption region c.

$$E = \hat{f} \times Z, \quad \text{(S4)}$$

$$E_{(m,r),(c,e)} = f_{m,r} \times z_{m,r}^{c,e}, \quad \text{(S5)}$$

where $E_{(m,r),(c,e)}$represents the mining-related biodiversity footprint associated with extracted commodity m, extraction region r, that is required to satisfy end-use sector e in consumption region c. The resulting impact matrix has dimensions (163 × 189) × (189 × 163), which can be further reconstructed into a 4D array with dimensions of 163 × 189 × 189 × 163.

The four dimensions should be interpreted as alternative attribution perspectives of the same total biodiversity footprint, rather than separate additive impact categories. Summing the four-dimensional array over all dimensions yields the global total mining-related biodiversity impact. For visualization and interpretation, each dimension was subsequently aggregated into broader categories. Extracted commodity sectors were aggregated into major mining commodities or commodity groups (Figure 5a), extraction and consumption regions were aggregated into the countries or regional groups (Figure 5b,c), and end-use sectors were aggregated into broader downstream sector groups (Figure 5d). Internationally displaced biodiversity footprints were calculated as the share of impacts for which the extraction region differed from the consumption region.

SI Results

Result S1. Local biodiversity intactness loss due to mining activities

Despite the similar spatial patterns shared by plants and animals in Figure 2a,b, the pressures contributing to the global mean species abundance (MSA) decline are different for the two species groups. For plant species, land use is considered the predominant pressure driving the global MSA decline. Therefore, the MSA loss of plant species in Figure 2b is proportional to the mining area in Figure S4. For animal species, multiple pressures, including land use, habitat fragmentation, and road disturbance, are taken into consideration when projecting the MSA changes. As shown in Figure S4 and Figure 2a, regions with larger mining areas do not necessarily show a more significant MSA loss of animal species. For example, although China ranks second in global mining areas (10%), it accounts for 6% of the mean global MSA decline in animals. Figure 3a shows the global mining area derived from Maus et al.[8] with the base map colored by natural land cover and human land use. For regions where mining activities are conducted more often in natural areas such as forests, grassland, and shrubland, the pressure of habitat fragmentation contributes more to the MSA loss compared to regions with mining activities around urban areas or secondary-vegetation land. For example, a large fraction of mining areas in Indonesia and Brazil are observed in natural regions while for China and South Africa, most of the mining activities are conducted in regions dominated by human land use (Figure 3a). It indicates that the risk of having fragmented natural patches due to mining activities tends to be higher in Indonesia and Brazil, contributing more to the MSA loss in those regions.

Result S2. Further examples on hotspots of global mining-related biodiversity loss

Indonesia, Brazil, and Peru experience notable local biodiversity intactness loss (measured as MSA in Figure 2a,b) due to the land use impact caused by mining activities and habitat fragmentation pressure, and at the same time, all these three countries are located in the regions with high biodiversity importance (measured as range rarity in Figure 2c,d). Although New Caledonia is not among the hotspots of local biodiversity intactness loss (Figure 2a,b) due to the limited mining areas, its high biodiversity importance due to the diverse habitats and high rates of endemic species (Figure 2c,d) leads to profound impacts on mining-related biodiversity loss (Figure 2e,f). In contrast, while Russia represents the largest share of global mining areas

(12%, Figure S4), mining activities there contribute to less than 1% of the global loss (not within the top countries in Figure 2e,f) in animal and plant biodiversity.

Result S3. Polygon-level biodiversity impacts across mining footprint size classes and region groups

To further explore heterogeneity among mining footprints represented in the updated global mining land-use dataset[8], we grouped all mining polygons into four footprint-size classes (i.e., <0.1, 0.1–1, 1–10, and >10 km^2) and analyzed their biodiversity impacts across size classes and region groups (Figure S6a,b,c). These size classes are used here as a spatial proxy for differences in mining footprint configuration and should not be interpreted as an explicit classification of artisanal and small-scale mining versus large-scale mining, because the underlying polygon dataset does not assign mine-type labels to individual polygons.

Figure S6a shows that biodiversity impacts are highly uneven across polygon size classes. Although polygons larger than 10 km^2 account for only 3.9% of all polygons, they contribute 64.7% of total mining area and 66.5% of total biodiversity impacts. By contrast, polygons smaller than 1 km^2 together represent 75.4% of all polygons, but contribute only 7.9% of total mining area and 5.2% of total impacts. Median biodiversity impact per polygon increases sharply with footprint size, indicating that cumulative biodiversity loss is dominated by a relatively small number of large mining footprints, whereas numerous small polygons create a more spatially dispersed impact pattern. This size-dependent pattern is also evident at the polygon level across region groups (Figure S6b). The scatter plot shows a clear positive relationship between polygon size and biodiversity impact in all 12 region groups. At the same time, polygons of similar size can differ in biodiversity impact by several orders of magnitude, especially in the small- to medium-size range. This spread shows that polygon size alone does not determine biodiversity burden and local ecological sensitivity, habitat fragmentation effects, and surrounding land-cover context also shape the impacts associated with a given footprint.

While Figure S6a,b focus on total biodiversity impact, Figure S6c addresses biodiversity impact intensity, defined as impact per unit mining area. This distinction is important because regions with large total impacts do not necessarily have the highest impacts per unit area. A prominent example is New Caledonia, which shows exceptionally high impact intensity across all size classes despite more moderate total mining area than major hotspot regions such as

Indonesia or Australia. More generally, most regions show a strong increase in impact intensity from the <0.1 km^2 class to the 0.1–1 km^2 class, followed by a weaker increase or near-stabilization in larger classes. However, this pattern is not universal. In China, Other North America, Europe, Brazil, Other Asia & Oceania, and Other South America, impact intensity peaks in the intermediate classes and then declines from 1–10 km^2 to >10 km^2.

Taken together, Figure S6 show that polygon size is a useful proxy for exploring heterogeneity in mining-related biodiversity impacts, but it does not fully explain either the total burden or the impact intensity of mining land use. Larger polygons dominate cumulative biodiversity loss, yet regional ecological context strongly modifies both absolute impacts and per-area impact intensity. Accordingly, this analysis should be interpreted as a first step toward differentiating broad mining footprint patterns within a global dataset that includes both large-scale mining and artisanal and small-scale mining, rather than as a formal mine-type comparison. A more explicit distinction between large-scale mining and artisanal and small-scale mining would require additional classification information, such as polygon morphology, clustering patterns, commodity context, or mine-level metadata.

Result S4. Global Mining Activities

Figure S4 shows the mining areas derived based on the dataset of Maus et al.[8] on a country level. For the total mining areas of 101,583 km^2, Russia (11.6%), China (10.2%), Australia (8.4%), the USA (8.1%), Indonesia (7.9%), and Brazil (5.8%) account for approximately half of the global mining land use. Apart from the top 15 countries listed in Figure S4, the remaining regions only add up to 20.5% of the global mining areas. Although Russia has the largest mining area, the amount of mining polygons in Russia is lower than that in China, Australia, and the USA, indicating that different regions might be characterized by different mining patterns, such as large-scale mining, and artisanal and small-scale mining.

The mining production data for coal, copper, gold, iron, lead, nickel, silver, and zinc were obtained from BGS[9] for the year of 2019 (Figure S7). China is the largest producer of coal, gold, lead, and zinc, accounting for 47%, 11%, 39%, and 30% of the global production, respectively. Chile is the biggest producer of copper, accounting for 28% of the global copper production, while Indonesia produces the highest amount of nickel, accounting for 39% of the global nickel production. Additionally, 58% of the iron ore is produced in Australia and China,

while 52% of the silver was produced in Mexico, Peru, and China. For detailed production of other mining commodities, see the complete dataset from World Mineral Statistics of BGS[9].

SI Discussion

Discussion S1. Global mining land use mapping

Mapping global mining areas is essential for biodiversity conservation, offering a strategic tool to identify overlaps between mining operations and ecologically sensitive territories and aiding in spatial planning of low-biodiversity-impact mining practices[8,10-13]. The progress in mapping global mining land use has been empowered by high-resolution earth observation data, manual delineation, and advanced machine learning algorithms (see recent mining land use datasets in Table S7). The global mining land use dataset[8] utilized in this study was updated based on the first version of the dataset[11], which included 21,060 polygons extending over 57,277 km^2 (approximately half the area of the updated version). It is worth noticing that the coverage of artisanal and small-scale mining was improved in the updated dataset and was nearly absent in the first version. Although the updated mining land-use dataset[8] used here includes both large-scale mining and artisanal and small-scale mining, the final polygon product does not assign mine-type labels to individual polygons. Accordingly, the present study cannot separate specific biodiversity footprints of large-scale mining and artisanal and small-scale mining, or supply-chain pathways on a polygon-by-polygon basis. The polygon-size analysis presented here should therefore be interpreted as a first step toward differentiating broad mining footprint patterns within a global dataset, while future research should combine polygon geometry with additional mine-level information, such as commodity context, clustering patterns, or site-specific metadata, to explicitly assess biodiversity impacts of large-scale mining and artisanal and small-scale mining. Although the two datasets from Maus et al.[8,11] might not be directly comparable since they were acquired based on different satellite data sources, at different times, and with distinct resolutions, applying the first version to this analysis might decrease the global overall biodiversity loss impacts caused by mining activities.

Additionally, the spatial distribution of global mining land use shows different patterns among different datasets, which would further change the relative contribution from each country to the global mining-related biodiversity loss impacts. For instance, in the updated dataset of Maus et al.[8], Russia accounted for the largest mining land use while it held fourth place in the first version of Maus et al.[11]. Additionally, the mining areas in Brazil, Peru, Guyana, Argentina, and Myanmar were more than tripled in the updated dataset[8], and were among the top 15 countries with the largest mining areas (Figure S4). Other mining land use datasets (e.g., mining land use map by Tang and Werner[10]) more strictly contour the edges of mining features

and disregard patches in between mines, leading to a smaller average area of each polygon compared to the dataset[8] used here. In our study, both mining features and patches in between were included as they are usually considered with a reduced ecological function due to landscape fragmentation[8,14-16]. Despite the high resolution of local biodiversity intactness (i.e., 10 arc seconds in this study) and global biodiversity importance (i.e., 30 arc seconds in this study), the model might still overlook certain mining sites if their delineated areas are significantly smaller than the model's resolution.

SI Figures

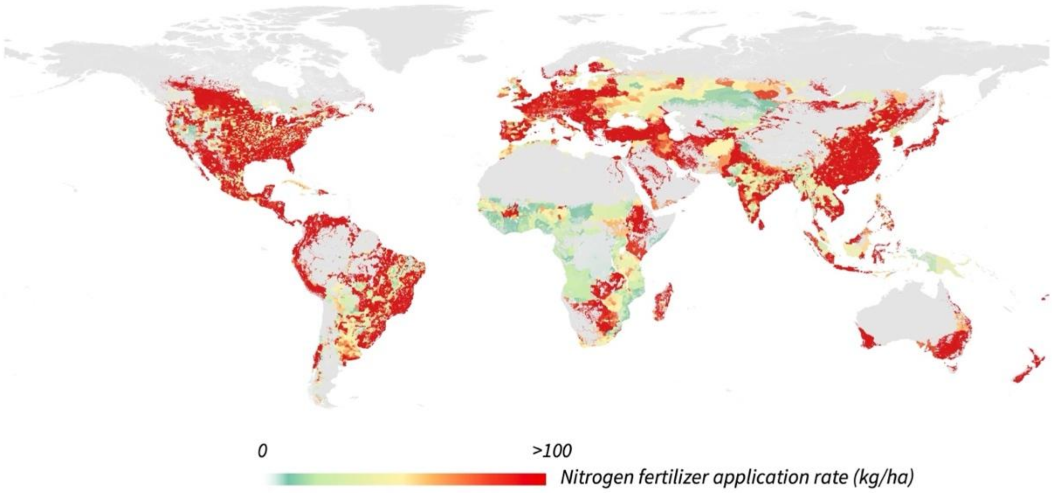

Figure S1. Global nitrogen fertilizer application rate. This map was conducted based on the global crop-specific nitrogen fertilization dataset of Adalibieke et al.[17], and was resampled using the nearest neighbor approach from its original resolution of 5-arc-minute to a finer 10-arc-second resolution to ensure consistency with the resolution of the land use land cover map. Global nitrogen fertilizer application rate was further utilized to identify the land use intensity of cropland areas. Blank regions in the map indicate non-available data. The land-use intensity classification was applied only to cropland, for which the GLOBIO 4 model distinguishes high-intensity and low-intensity cropland classes. By contrast, no additional intensity classification was applied to forestry or pasture-related land use, because the GLOBIO 4 parameterization used here does not provide a comparable intensity split for forestry, while grazing-related land use is already represented by separate classes (grazing, pasture, and rangeland) with distinct MSA values. In addition, when modeling fragmentation-induced MSA loss with GLOBIO 4, cropland and pasture are treated as human land use categories regardless of within-class intensity (Table S3), so further subdivision of these classes would not have changed that part of the calculation.

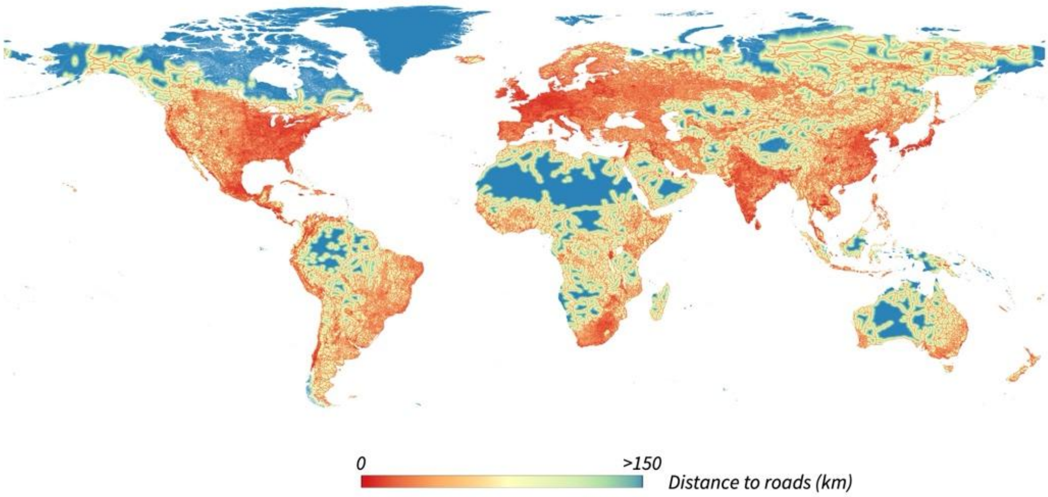

Figure S2. Global distribution map of distance to roads. Spatial data on road infrastructures are obtained from the Global Roads Inventory Project (GRIP)[18]. Primary and secondary roads (i.e., road types 1, 2, and 3 in the GRIP database) are considered when calculating habitat fragmentation and road disturbance impacts. Blank regions in the map indicate non-available data.

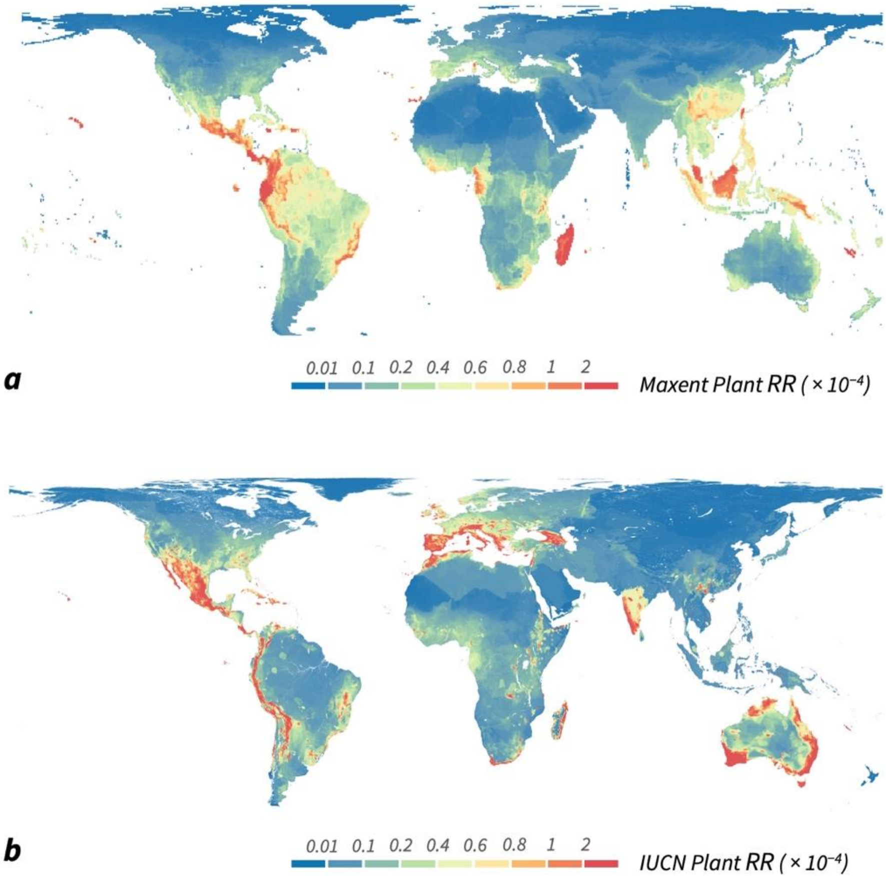

Figure S3. Global spatial distribution of plant biodiversity importance expressed as range rarity. (a) Range rarity (RR) based on the range maps from Borgelt et al.[19], covering 27,208 plant species. (**b**) Range rarity based on the range maps from IUCN[20], covering 5,395 plant species. The range rarity of plants derived from Borgelt[19] was resampled from a 30-arc-minute resolution to a 30-arc-second resolution to align with the range rarity of animals. Blank regions in each map indicate non-available data.

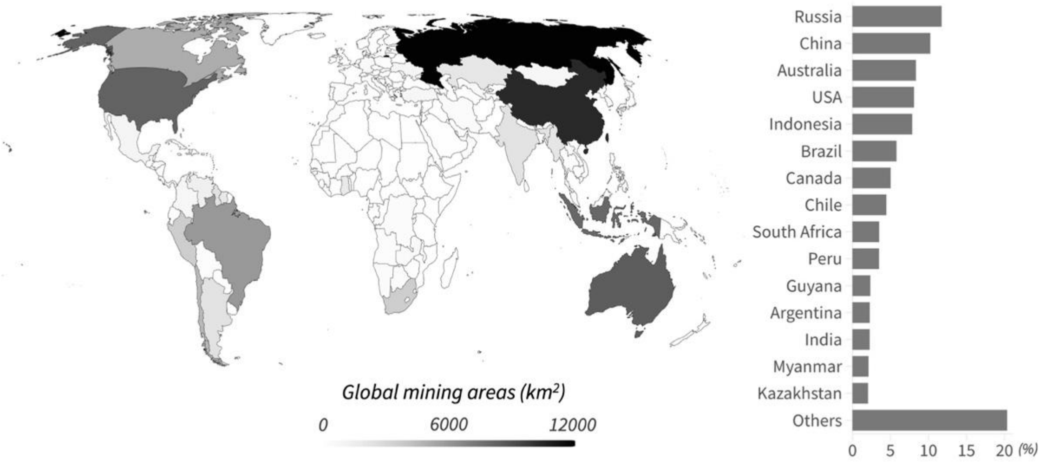

Figure S4. Global mining land use areas by country. The map was derived based on the dataset of Maus et al.[8] (in total 101,583 km^2). The contributions from the top 15 countries to the global mining areas are listed together with the spatial distribution maps in descending order. Abbreviation: USA, United States of America.

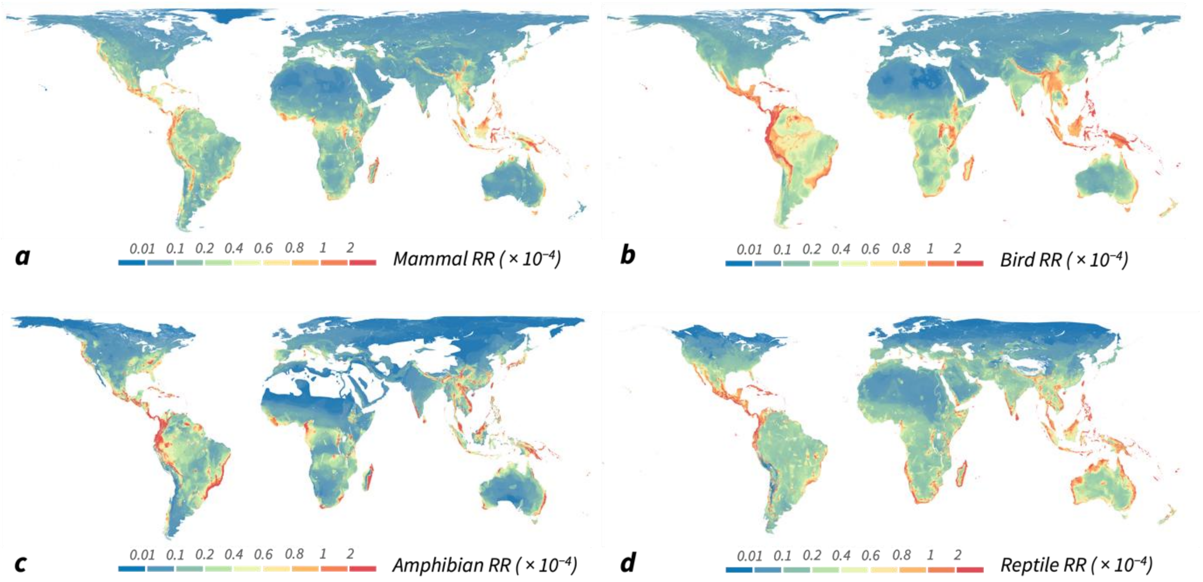

Figure S5. The spatial distribution of the global biodiversity importance of animal taxa. Range rarity (RR) was calculated at 30-arc-second resolution to represent global biodiversity importance for each grid cell of (**a**) terrestrial mammals, (**b**) birds, (**c**) amphibians, and (**d**) terrestrial reptiles. The range rarity of four animal taxa (**a**–**d**) was further aggregated to the overall animal range rarity in Figure 2c.

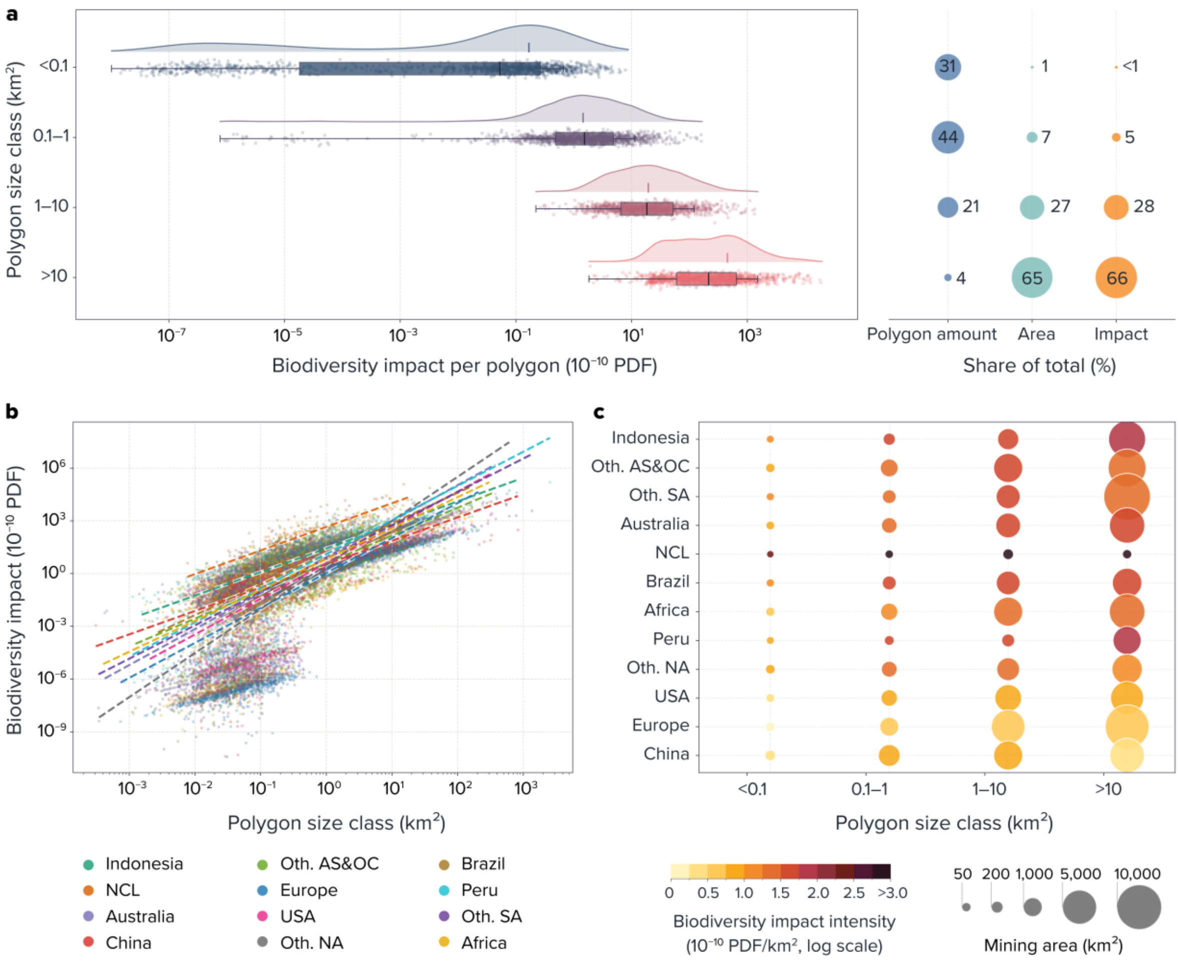

Figure S6. Polygon-level heterogeneity in mining-related biodiversity impacts across mining footprint size classes and region groups. **a**, The distribution of biodiversity impact per polygon and the corresponding shares of total polygon count, total mining area, and total biodiversity impact are compared across four mining footprint size classes (i.e., <0.1, 0.1–1, 1–10, and >10 km^2). **b**, The relationship between polygon size and biodiversity impact is analyzed at the individual polygon level. Each point is colored by region group, and dashed lines show region-specific log-log fitted relationships. Both axes are displayed on logarithmic scales. **c**, The variation of biodiversity impact intensity is analyzed across region groups and polygon size classes. Circle size indicates total mining area and circle color indicates biodiversity impact intensity. Abbreviations: PDF, potentially disappeared fraction; USA, United States of America; Oth. AS&OC, Other Asia & Oceania; Oth. SA, Other South America; NCL, New Caledonia; NA, Other North America.

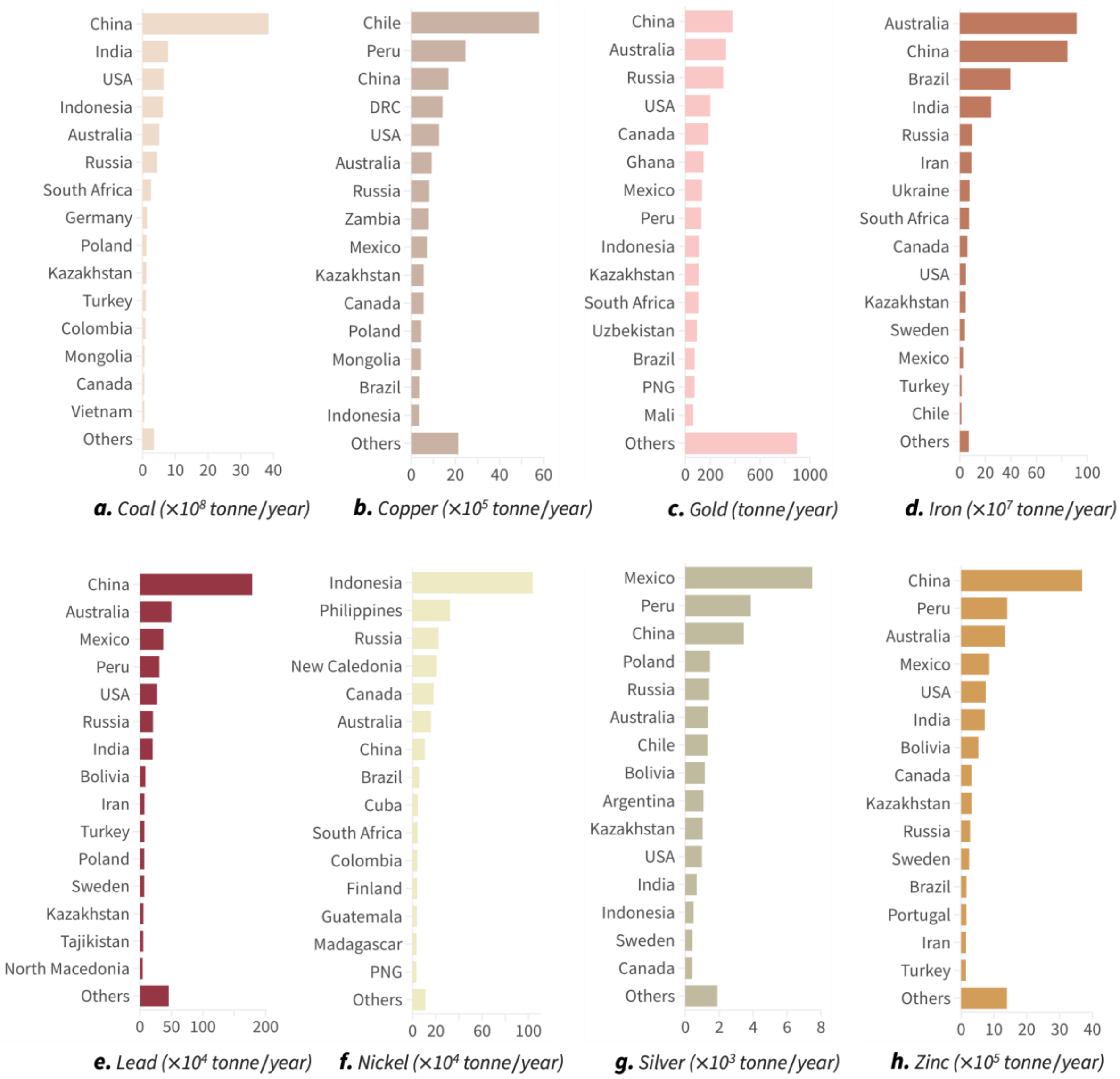

Figure S7. Global mining production data. Mining production data for the year 2019 based on the World Mineral Statistics of BGS[9] for **a.** coal, **b.** copper, **c.** gold, **d.** iron, **e.** lead, **f.** nickel, **g.** silver, and **h.** zinc. For the detailed production of remaining mining commodities, see details from the complete dataset of BGS[9]. Abbreviation: USA, United States of America; DRC, Democratic Republic of the Congo; PNG, Papua New Guinea.

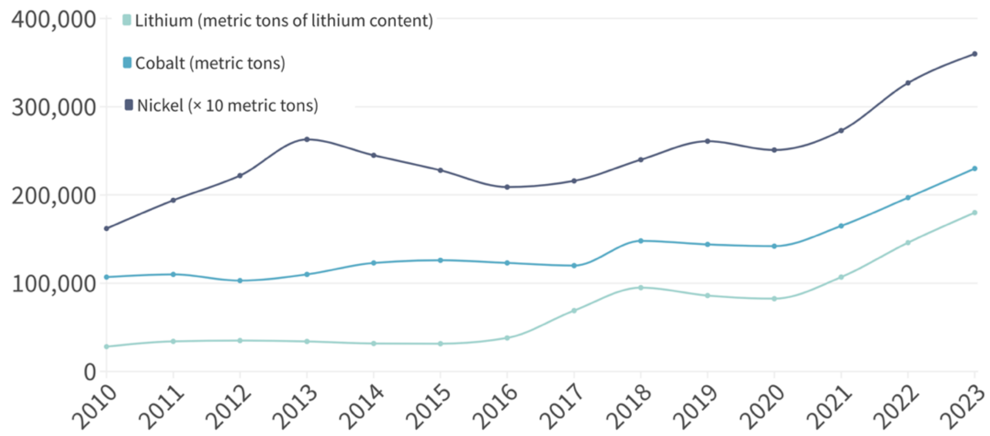

Figure S8. Production data for energy transition minerals, including lithium, cobalt, and nickel from 2010 to 2023 based on U. S. Geological Survey[21].

SI Tables

Table S1. The GLOBIO 4 land use land cover code and the corresponding description. Land use land cover classes with asterisks are not present in the land use map developed by GLOBIO 4 model[22]. The mining class (i.e., code 8) was classified using mining land use data from Maus et al.[8], and the highest priority was accorded during the land use land cover allocation process. General cropland (i.e., code 10) was further classified into intensive and minimal cropland (i.e., codes 11 and 12) based on the nitrogen fertilizer application rate (Figure S1).

GLOBIO 4 Code	GLOBIO 4 LULC description
0	No data/Undefined
1	Urban settlement
8*	Mining
10	Cropland
11*	Cropland intensive use
12*	Cropland minimal use
13	Mosaic cropland (>50%) / natural vegetation (tree shrub herbaceous cover) (<50%)
14	Mosaic natural vegetation (tree shrub herbaceous cover) (>50%) / cropland (<50%)
20	Grazing
30	Forestry
40	Secondary vegetation
50	Tree cover broadleaved evergreen closed to open (>15%)
60	Tree cover broadleaved deciduous closed to open (>15%)
61	Tree cover broadleaved deciduous closed (>40%)
62	Tree cover broadleaved deciduous open (15-40%)
70	Tree cover needleleaved evergreen closed to open (>15%)
71	Tree cover needleleaved evergreen closed (>40%)
72	Tree cover needleleaved evergreen open (15-40%)
80	Tree cover needleleaved deciduous closed to open (>15%)
81	Tree cover needleleaved deciduous closed (>40%)
82	Tree cover needleleaved deciduous open (15-40%)
90	Tree cover mixed leaf type (broadleaved and needleleaved)
100	Mosaic tree and shrub (>50%) / herbaceous cover (<50%)
110	Mosaic herbaceous cover (>50%) / tree and shrub (<50%)
120	Shrubland
121	Shrubland evergreen
122	Shrubland deciduous
130	Grassland
140	Lichens and mosses
150	Sparse vegetation (tree shrub herbaceous cover) (<15%)
151	Sparse tree (<15%)
152	Sparse shrub (<15%)
153	Sparse herbaceous cover (<15%)
160	Tree cover flooded fresh or brakish water
170	Tree cover flooded saline water
180	Shrub or herbaceous cover flooded fresh/saline/brakish water
200	Bare areas
201	Consolidated bare areas
202	Unconsolidated bare areas
210	Water bodies
220	Permanent snow and ice

Table S2. The GLOBIO 4 land use land cover code and the corresponding mean species abundance (MSA) value for animals and plants.

GLOBIO 4 Code	GLOBIO 4 LULC description	MSA Animals	MSA Plants
1	Urban	0.264	0.361
8	Mining	0.264	0.361
10	Cropland	0.452	0.156
11	Cropland – high intensity	0.401	0.149
12	Cropland – low intensity	0.502	0.162
13	Mosaic cropland (>50%) / natural vegetation (tree shrub herbaceous cover) (<50%)	0.671	0.493
14	Mosaic natural vegetation (tree shrub herbaceous cover) (>50%) / cropland (<50%)	0.781	0.662
20	Grazing	0.452	0.156
21	Pasture	0.401	0.149
22	Rangeland	0.502	0.162
30	Forestry	0.589	0.259
40	Secondary vegetation	0.621	0.568
50	Tree cover broadleaved evergreen closed to open (>15%)	1	1
60	Tree cover broadleaved deciduous closed to open (>15%)	1	1
61	Tree cover broadleaved deciduous closed (>40%)	1	1
62	Tree cover broadleaved deciduous open (15-40%)	1	1
70	Tree cover needleleaved evergreen closed to open (>15%)	1	1
71	Tree cover needleleaved evergreen closed (>40%)	1	1
72	Tree cover needleleaved evergreen open (15-40%)	1	1
80	Tree cover needleleaved deciduous closed to open (>15%)	1	1
81	Tree cover needleleaved deciduous closed (>40%)	1	1
82	Tree cover needleleaved deciduous open (15-40%)	1	1
90	Tree cover mixed leaf type (broadleaved and needleleaved)	1	1
100	Mosaic tree and shrub (>50%) / herbaceous cover (<50%)	1	1
110	Mosaic herbaceous cover (>50%) / tree and shrub (<50%)	1	1
120	Shrubland	1	1
121	Shrubland evergreen	1	1
122	Shrubland deciduous	1	1
130	Grassland	1	1
140	Lichens and mosses	1	1
150	Sparse vegetation (tree shrub herbaceous cover) (<15%)	1	1
151	Sparse tree (<15%)	1	1
152	Sparse shrub (<15%)	1	1
153	Sparse herbaceous cover (<15%)	1	1
160	Tree cover flooded fresh or brakish water	1	1
170	Tree cover flooded saline water	1	1
180	Shrub or herbaceous cover flooded fresh/saline/brakish water	1	1
200	Bare areas	1	1
201	Consolidated bare areas	1	1
202	Unconsolidated bare areas	1	1
210	Water bodies	1	1
220	Permanent snow and ice	1	1

Table S3. The identification of nature land cover and human land use based on the GLOBIO 4 land use land cover code.

LULC description	GLOBIO 4 Code
Nature land cover	22, 30, 40, 50, 60, 61, 62, 70, 71, 72, 80, 81, 82, 90, 100, 110, 120, 121, 122, 130, 140, 150, 151, 152, 153, 160, 170, 180, 200, 201, 202, 220
Human land use	1, 8, 10, 11, 12, 13, 14, 20, 21

Table S4. The land use land cover code of the potential natural vegetation (PNV) map and the corresponding description. This PNV map developed by Hengl et al.[23], which delineates the expected land cover classes in the absence of human activities, was utilized as a natural reference to assess the impact of mining activities on local biodiversity intactness.

PNV Code	PNV LULC description	PNV aggregated LULC description
0	Unknown	Unknown
20	Shrubs	Shrubs
21	Sub-polar or polar barren-lichen-moss, grassland	Shrubs
30	Herbaceous vegetation	Herbaceous vegetation
40	Cropland	Cropland
50	Urban/built-up	Urban/built-up
60	Bare/sparse vegetation	Bare/sparse vegetation
70	Snow and ice	Snow and ice
80	Permanent water bodies	Permanent water bodies
90	Herbaceous wetland	Herbaceous wetland
100	Moss and lichen	Moss and lichen
111	Closed forest, evergreen needleleaf	Forests
112	Closed forest, evergreen broadleaf	Forests
113	Closed forest, deciduous needleleaf	Forests
114	Closed forest, deciduous broadleaf	Forests
115	Closed forest, mixed	Forests
116	Closed forest, unknown	Forests
121	Open forest, evergreen needleleaf	Forests
122	Open forest, evergreen broadleaf	Forests
123	Open forest, deciduous needleleaf	Forests
124	Open forest, deciduous broadleaf	Forests
125	Open forest, mixed	Forests
126	Open forest, unknown	Forests
127	Subtropical/tropical mangrove vegetation	Forests
200	Open sea	Open sea

Table S5. The reference for correlating the land use land cover categories of the potential natural vegetation (PNV) with those used in the GLOBIO 4 model.

PNV Code	PNV LULC description	GLOBIO 4 code	GLOBIO 4 LULC description
0	Unknown	0	No data/Undefined
20	Shrubs	120	Shrubland
21	Sub-polar or polar barren-lichen-moss, grassland	120	Shrubland
30	Herbaceous vegetation	110	Mosaic herbaceous cover (>50%) / tree and shrub (<50%)
40	Cropland	10	Cropland
50	Urban/built-up	1	Urban-settlement
60	Bare/sparse vegetation	200	Bare areas
70	Snow and ice	220	Permanent snow and ice
80	Permanent water bodies	210	Water bodies
90	Herbaceous wetland	180	Shrub or herbaceous cover flooded fresh/saline/brackish water
100	Moss and lichen	140	Lichens and mosses
111	Closed forest, evergreen needleleaf	71	Tree cover needleleaved evergreen closed (>40%)
112	Closed forest, evergreen broadleaf	50	Tree cover broadleaved evergreen closed to open (>15%)
113	Closed forest, deciduous needleleaf	81	Tree cover needleleaved deciduous closed (>40%)
114	Closed forest, deciduous broadleaf	61	Tree cover broadleaved deciduous closed (>40%)
115	Closed forest, mixed	90	Tree cover mixed leaf type (broadleaved and needleleaved)
116	Closed forest, unknown	90	Tree cover mixed leaf type (broadleaved and needleleaved)
121	Open forest, evergreen needleleaf	72	Tree cover needleleaved evergreen open (15-40%)
122	Open forest, evergreen broadleaf	50	Tree cover broadleaved evergreen closed to open (>15%)
123	Open forest, deciduous needleleaf	82	Tree cover needleleaved deciduous open (15-40%)
124	Open forest, deciduous broadleaf	62	Tree cover broadleaved deciduous open (15-40%)
125	Open forest, mixed	90	Tree cover mixed leaf type (broadleaved and needleleaved)
126	Open forest, unknown	90	Tree cover mixed leaf type (broadleaved and needleleaved)
127	Subtropical/tropical mangrove vegetation	90	Tree cover mixed leaf type (broadleaved and needleleaved)
200	Open sea	210	Water bodies

Table S6. Per-capita consumption-based mining-related biodiversity loss footprint among global regions in 2019. Countries or regions that are listed as consumption regions in Figure 5 are country-specific in the per-capita consumption-based analysis. The remaining countries are aggregated into world regions or subregions according to the Standard Country or Area Codes for Statistical Use (M49) from UNSD[24].

Region	Population (million capita)	Biodiversity impacts (global PDF)	Global share	Per-capita biodiversity impacts (global PDF/capita)	Compared to global average
China	1,416	1.1E-04	31.4%	7.4E-14	171%
USA	328	2.8E-05	8.4%	8.6E-14	198%
Japan	127	2.9E-05	8.8%	2.3E-13	533%
Indonesia	270	1.7E-05	5.1%	6.3E-14	145%
India	1,383	1.8E-05	5.3%	1.3E-14	30%
Australia	25	7.4E-06	2.2%	2.9E-13	668%
Europe	745	3.3E-05	9.7%	4.4E-14	100%
South America	428	2.7E-05	8.1%	6.3E-14	145%
Other North America	251	1.4E-05	4.0%	5.4E-14	124%
Africa	1322	1.9E-05	5.6%	1.4E-14	33%
Other Asia & Oceania	1,411	3.8E-05	11.4%	2.7E-14	62%
**Total population (million capita)**	7,706	**Total biodiversity impacts (global PDF)**	3.4E-04	**Global average per-capita biodiversity impacts (global PDF/capita)**	4.4E-14

Table S7. Comparison of different mining land use datasets. Abbreviation: LSM, large-scale mining; ASM, artisanal and small-scale mining.

Polygons/mines	Area (km^2)	Included features	Patches included	Reference
44,929 polygons	101,583	LSM & ASM: open cuts, tailing dams, waste rock dumps, water ponds, processing plants, and other ground features related to the mining activities	Yes	[8]
21,060 polygons	57,277	Mainly LSM: open cuts, tailing dams, waste rock dumps, water ponds, and processing infrastructure	Yes	[11]
74,548 polygons	65,585	LSM & ASM: dumps, pits, water ponds, tailings dams, heap leach pads and processing/milling infrastructure	No	[10]
295 mines	3,633	LSM: open pits, waste rock dumps, water ponds, tailings storage facilities, heap leach pads, milling infrastructure and other features	No	[12]
24,605 polygons	31,396	LSM & ASM: open cut pits, milling infrastructure, waste rock dumps, and tailings storage facilities	No	[13]

REFERENCES

1. Cabernard, L., Pfister, S. & Hellweg, S. Resolved Exiobase version 3 (REX3) (3.2) *Zenodo* (2024).
2. Cabernard, L. & Pfister, S. A highly resolved MRIO database for analyzing environmental footprints and Green Economy Progress. *Science of The Total Environment* **755**, 142587 (2021).
3. Cabernard, L. & Pfister, S. Hotspots of Mining-Related Biodiversity Loss in Global Supply Chains and the Potential for Reduction through Renewable Electricity. *Environmental Science & Technology* **56**, 16357-16368 (2022).
4. Cabernard, L., Pfister, S. & Hellweg, S. A new method for analyzing sustainability performance of global supply chains and its application to material resources. *Science of The Total Environment* **684**, 164-177 (2019).
5. Cabernard, L., Pfister, S. & Hellweg, S. Biodiversity impacts of recent land-use change driven by increases in agri-food imports. *Nature Sustainability* **7**, 1512-1524 (2024).
6. Santero, N. & Hendry, J. Harmonization of LCA methodologies for the metal and mining industry. *The International Journal of Life Cycle Assessment* **21**, 1543-1553 (2016).
7. Nuss, P. & Eckelman, M.J. Life Cycle Assessment of Metals: A Scientific Synthesis. *PLOS ONE* **9**, e101298 (2014).
8. Maus, V., Giljum, S., da Silva, D.M., Gutschlhofer, J., da Rosa, R.P., Luckeneder, S., Gass, S.L.B., Lieber, M. & McCallum, I. An update on global mining land use. *Scientific Data* **9**, 433 (2022).
9. BGS. World mineral statistics data. *https://www.bgs.ac.uk/mineralsuk/statistics/world-mineral-statistics/world-mineral-statistics-data-download/ [Accessed: February 2024]* (2024).
10. Tang, L. & Werner, T.T. Global mining footprint mapped from high-resolution satellite imagery. *Communications Earth & Environment* **4**, 134 (2023).
11. Maus, V., Giljum, S., Gutschlhofer, J., da Silva, D.M., Probst, M., Gass, S.L.B., Luckeneder, S., Lieber, M. & McCallum, I. A global-scale data set of mining areas. *Scientific Data* **7**, 289 (2020).
12. Werner, T.T., Mudd, G.M., Schipper, A.M., Huijbregts, M.A.J., Taneja, L. & Northey, S.A. Global-scale remote sensing of mine areas and analysis of factors explaining their extent. *Global Environmental Change* **60**, 102007 (2020).
13. Liang, T., Werner, T.T., Heping, X., Jingsong, Y. & Zeming, S. A global-scale spatial assessment and geodatabase of mine areas. *Global and Planetary Change* **204**, 103578 (2021).
14. Montibeller, B., Kmoch, A., Virro, H., Mander, Ü. & Uuemaa, E. Increasing fragmentation of forest cover in Brazil's Legal Amazon from 2001 to 2017. *Scientific Reports* **10**, 5803 (2020).
15. Kuipers, K.J.J., May, R. & Verones, F. Considering habitat conversion and fragmentation in characterisation factors for land-use impacts on vertebrate species richness. *Science of The Total Environment* **801**, 149737 (2021).
16. Kuipers, K.J.J., Hilbers, J.P., Garcia-Ulloa, J., Graae, B.J., May, R., Verones, F., Huijbregts, M.A.J. & Schipper, A.M. Habitat fragmentation amplifies threats from habitat loss to mammal diversity across the world's terrestrial ecoregions. *One Earth* **4**, 1505-1513 (2021).
17. Adalibieke, W., Cui, X., Cai, H., You, L. & Zhou, F. Global crop-specific nitrogen fertilization dataset in 1961–2020. *Scientific Data* **10**, 617 (2023).
18. Meijer, J.R., Huijbregts, M.A., Schotten, K.C. & Schipper, A.M. Global patterns of current and future road infrastructure. *Environmental Research Letters* **13**, 064006 (2018).
19. Borgelt, J., Sicacha-Parada, J., Skarpaas, O. & Verones, F. Native range estimates for red-listed vascular plants. *Scientific Data* **9**, 117 (2022).
20. IUCN. The IUCN Red List of Threatened Species. Version 2023-12. *https://www.iucnredlist.org. [Accessed: December 2023]* (2023).
21. U. S. Geological Survey. Mineral Commodity Summaries 2024. *Mineral Commodity Summaries*, 212 (2024).

22. GLOBIO. GLOBIO4 scenario data. *https://www.globio.info/globio-data-downloads*. *[Accessed: December 2023]* (2024).

23. Hengl, T., Jung, M. & Visconti, P. Potential distribution of land cover classes (Potential Natural Vegetation) at 250 m spatial resolution. *Zenodo* *https://doi.org/10.5281/zenodo.3631254* (2020).

24. UNSD. Standard Country or Area Codes for Statistical Use (M49). *https://unstats.un.org/unsd/methodology/m49/* *[October 2024]* (2024).
